# A novel bacteriophage cocktail reduces and disperses *P* *seudomonas aeruginosa* biofilms under static and flow conditions

**DOI:** 10.1111/1751-7915.12316

**Published:** 2015-09-08

**Authors:** Diana R. Alves, P. Perez‐Esteban, W. Kot, J.E. Bean, T. Arnot, L.H. Hansen, Mark C. Enright, A. Tobias A. Jenkins

**Affiliations:** ^1^ Department of ChemistryUniversity of BathBathClaverton DownBA2 7AYUK; ^2^ Department of Chemical EngineeringUniversity of BathBathClaverton DownBA2 7AYUK; ^3^ Department of Environmental ScienceAarhus UniversitetFrederiksborgvej 399, Postboks, 358RoskildeDK‐4000Denmark; ^4^ School of Healthcare SciencesManchester Metropolitan UniversityJohn Dalton Building, Chester StreetManchesterM1 5GDUK

## Abstract

*P* *seudomonas aeruginosa* is an opportunistic human pathogen that forms highly stable communities – biofilms, which contribute to the establishment and maintenance of infections. The biofilm state and intrinsic/acquired bacterial resistance mechanisms contribute to resistance/tolerance to antibiotics that is frequently observed in *P* *. aeruginosa* isolates. Here we describe the isolation and characterization of six novel lytic bacteriophages: viruses that infect bacteria, which together efficiently infect and kill a wide range of *P* *. aeruginosa* clinical isolates. The phages were used to formulate a cocktail with the potential to eliminate *P* *. aeruginosa* PAO1 planktonic cultures. Two biofilm models were studied, one static and one dynamic, and the phage cocktail was assessed for its ability to reduce and disperse the biofilm biomass. For the static model, after 4 h of contact with the phage suspension (MOI 10) more than 95% of biofilm biomass was eliminated. In the flow biofilm model, a slower rate of activity by the phage was observed, but 48 h after addition of the phage cocktail the biofilm was dispersed, with most cells eliminated (> 4 logs) comparing with the control. This cocktail has the potential for development as a therapeutic to control *P* *. aeruginosa* infections, which are predominantly biofilm centred.

## Introduction

*Pseudomonas aeruginosa* are waterborne, highly versatile bacteria with minimal growth requirements that are ubiquitous in the environment (Frimmersdorf *et al*., 2010). Although, not part of the normal human flora, their versatility allows them to become serious and occasionally fatal pathogenic agents, if the opportunity for infection and growth is presented. The bacteria are often found colonizing hospital settings and medical devices, such as indwelling catheters (Gilsdorf *et al*., 1989). Immunocompromised patients are commonly colonized, and this can lead to chronic pulmonary infections in cystic fibrosis (CF) patients, and skin and soft tissue infections of severe burn victims that can ultimately lead to bacteraemia. Apart from their versatility, two additional factors make these bacteria directly on the top of the most problematic and challenging bacterial pathogens to treat. *Pseudomonas aeruginosa* cells commonly form biofilm communities, especially when infecting wounds or the lungs; here the infection establishes more effectively and resilience to environmental stresses is higher (Drenkard, 2003). Second, the incidence of *P. aeruginosa* antibiotic‐resistant isolates has increased significantly over the past two decades (Lister *et al*., 2009) and the species has acquired resistance to every licensed antibiotic class (Strateva and Yordanov, 2009). Inappropriate antibiotic use has selected for resistance in a species with a high recombination rate and exceptional genomic diversity (Kos *et al*., 2015). In addition, defects in the *P. aeruginosa* methyl‐directed mismatch repair (MMR) system make mutation recognition and repair less effective, leading to hypermutability. In fact, it was reported in a study in CF patients suffering from a chronic *P. aeruginosa* infection that 36% of the isolates showed a hypermutability phenotype (Oliver *et al*., 2000). For this reason, alternatives to antimicrobial drugs are being investigated to treat biofilm infections caused by antibiotic‐resistant *P. aeruginosa* isolates. Bacteriophages (phages) are a possible alternative to antibiotic drugs, with several potential advantages. Phages are natural predators of bacteria and phage infection and killing of bacteria has a completely different mode of action to antibiotics. Consequently, antibiotic‐resistant bacteria present no special challenge to phage therapy (Mann, 2008; Kutter *et al*., 2010). Phages are versatile agents that can be used in liquid, impregnated into hydrogels or solids, and are hence suitable not only for use by most delivery routes, such as topical application and inhalation, but also oral and parental delivery (Matsuzaki *et al*., 2005; Bean *et al*., 2014; Esteban *et al*., 2014). An additional advantage of phage already with promising results (Fu *et al*., 2010; Pires *et al*., 2011; Alves *et al*., 2014) is their ability to disrupt and eradicate biofilms (for review see Harper *et al*., 2014): (i) phage amplification allows access to the cells buried deeper in the biofilm; (ii) phages can carry or express depolymerizing enzymes that degrade the inter‐cellular polysaccharide matrix present in biofilm; (iii) if not coding for a depolymerase enzyme, the phages can induce expression of depolymerases from within the host genome (Bartell and Orr, 1969); (iv) ‘persister’ cells can still be infected by phages, although no proliferation will occur until these cells are ‘awakened’, after which phage replication will take place and burst the cell (Pearl *et al*., 2008). Here we describe not only the isolation and characterization of six novel phages against clinical strains of *P. aeruginosa*, but also we assess their antimicrobial efficacy in planktonic and in static and flow biofilm models.

## Results

### Phage cocktail isolation and host range determination

Phages were isolated from sewage and flood water samples after enrichment with *P. aeruginosa* strains of diverse clinical isolates, obtained from both acute and chronic clinical situations, listed in Table 1. Six phages showing clear isolated plaques surrounded by halos were selected. Halos are caused by the diffusion of enzymatic molecules and are usually an indication of a phage capable of displaying enzymatic activity towards the polysaccharide matrix of biofilms (Hughes *et al*., 1998). Lytic phages were evaluated with respect to their host range of infectivity. Phages were tested against *P. aeruginosa* isolates in order to select those with broad host ranges. Six of the 11 phages tested were selected to establish a phage cocktail, as their combination gave the highest percentage of coverage against the target pathogens (Table 2). Hence, the selected phages – DL52, DL54, DL60, DL62, DL64 and DL68 – gave a total coverage of 90% [95% confidence interval (CI), 62% to 94%; 18 isolates out of 22 were susceptible].

**Table 1:** List of human bacterial *P* *. aeruginosa* strains isolated from acute and chronic sites of infection, with their respective country of origin and related information

	Isolate	ST	Country	Source
	PAO1	–	Standard Laboratory Reference Strain	(250)
Acute infections	PA45311	–	Bristol, UK	n.d.
	PA45291	–	Bristol, UK	n.d.
	PA45235	–	Bristol, UK	n.d.
	PA45379	–	Bristol, UK	n.d.
	PA45321	–	Bristol, UK	n.d.
Chronic infections	BC00918	–	Genova , Italy	CF, Male, 23
	BC01026	–	Australia	n.d.
	BC00909	–	Paris, France	CF, Male, 25
	BC00888	1517	Utrecht, The Netherlands	CF, M, 11/01/2007
	BC00907	1528	Paris, France	CF, Female, 24
	BC00917	1544	Genova , Italy	CF, Male, 29 years
	BC00920	1547	Lisbon, Portugal	CF, Male, 32 years
	BC00921	1535	Lisbon, Portugal	CF, Female, 37 Years
	BC00922	155	Lisbon, Portugal	CF, Female, 8 Years
	BC00923	1548	Lisbon, Portugal	CF, Female, 19 years
	BC00931	–	Bordeaux, France	CF, Female
	BC00932	1529	Bordeaux, France	CF, Male
	BC00933	1530	Bordeaux, France	CF, Female
	BC00934	1552	Bordeaux, France	CF, Male
	BC00935	–	Bordeaux, France	CF, Male

CF, cystic fibrosis; n.d., no data available.

**Table 2:** Sensitivity screening of phage cocktail against *P* *. aeruginosa* clinical isolates

*Pseudomonas aeruginosa* isolates	DL52	DL54	DL60	DL62	DL64	DL68	Cocktail
BC00932	I	I	I	S	I	S	S
BC00931	I	R	I	I	I	S	S
BC00923	R	I	I	R	R	R	I
PAO1	S	S	S	S	S	S	S
BCO1O26	I	I	R	S	S	S	S
BC00935	S	S	S	I	S	S	S
BC00934	R	S	S	I	I	S	S
PAB45379	I	I	R	S	R	I	S
BC00907	S	S	S	S	S	S	S
PAB45235	R	R	R	I	S	I	S
PAB45321	R	R	I	R	R	R	I
PAB45291	S	S	S	S	S	S	S
BCOO909	R	R	R	S	R	I	S
BC00918	R	R	R	S	R	R	S
BC00917	R	I	R	I	S	S	S
BC00920	S	S	S	I	S	S	S
BC00921	R	R	I	R	R	R	I
PAB45311	I	R	R	R	R	R	I
BC00922	I	I	R	S	S	I	S
BC00888	I	I	R	R	S	S	S
BC00933	S	S	I	R	S	S	S
BC00920	I	R	I	S	S	S	S
Hit %	30	35	30	50	60	65	90

Bacterial isolates were susceptible (clear spot; S; green), intermediate (turbid spot; I; yellow), or resistant (no disturbance of bacterial lawn; R; red) to phage infection.

### Morphology of phage particles

**Figure 1:**
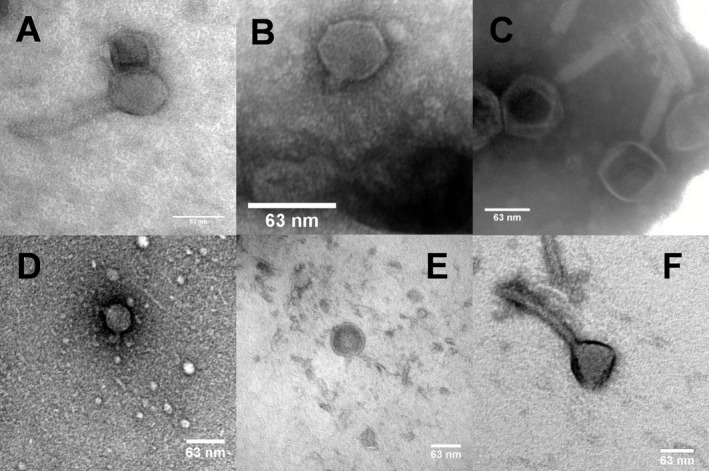
Electron micrograph images of phage (A) DL52, (B) DL54, (C) DL60, (D) DL62, (E) DL64 and (F) DL68 infecting *P* *. aeruginosa* negatively stained with 1% uranyl acetate. Scale is indicated by the bars.

The six *P. aeruginosa* phages were characterized with respect to their morphology under transmission electron microscopy. Two different types of morphology were identified, as shown in Fig. 1. DL52, DL60 and DL68 phages posse an icosahedral head of ∼ 60 nm, ∼ 76 nm and ∼ 72 nm in diameter respectively. Long contractile tails, with tail fibres, of approximately ∼ 123 × 18 nm, ∼ 95 × 19 nm and ∼ 177 × 23 nm, respectively, were also observed. Phages DL54, DL62 and DL64 have an icosahedral head of approximately ∼ 45 nm, ∼ 63 nm and ∼ 65 nm in diameter, respectively, and a short tail of ∼ 12 × 9 nm and ∼ 13 × 9 nm with no tail fibres being visible. For DL64 the tail was clearly visible; however, fibres protruding from the capsid are observed. According to the system of Ackermann classification (Ackermann, 2006) such morphology shows the first group of phages as belonging to the *Myoviridae* family and the second group as belonging to the *Podoviridae* family, both from the order *Caudovirales*. The samples of the observed *Myoviridae* phages showed the presence of many disintegrated phages, where several free capsids and free tails could be observed. Samples of phages DL54 and DL60 showed the presence of phage aggregates.

### Phage genomic characterization

**Figure 2:**
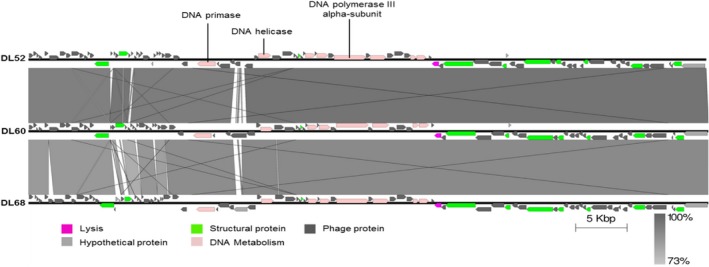
Comparative genomic analysis of the annotated *M* *yoviridae* phages DL52, DL60 and DL68. Nucleotide sequences were compared using the Artemis Comparison Tool (ACT). Predicted reading frames are denoted by arrows and genes encoding proteins with at least 73% amino acid identity between the genomes are indicated by shaded regions.

DNA from six *P. aeruginosa* phages was extracted and genome sequencing was performed. All six phages seem to have a linear dsDNA genome with terminal redundancy, suggesting that they have a headful packaging system (Streisinger *et al*., 1967). The genome sizes observed for phages DL52, DL60 and DL68 comprised 65 867 bp, 66 103 bp and 66 111 bp respectively. Figure 2 shows a comparison between these *Myoviridae* phages, performed using the blastn algorithm (Madden, 2003). The phages share 90% of their nucleotide sequence and in particular DL52 and DL60 are 96% identical. Genes are arranged in a typically compact manner with a coding percentage ranging from 89.7% to 91.6% and tRNA genes are absent from all of the phages. *Ps aeruginosa* shows a G + C% content of 66.6% and for all phages their G + C% content was lower (between 52.4% and 62.2%). Phages DL52 and DL60 show strong genomic similarities to phage PB1 (> 96%) and phage LBL3 (> 92%), which have recently been sequenced (Ceyssens *et al*., 2009). Phage DL68 shares high similarities with phage KPP12 DNA (96%) (Fukuda *et al*., 2012), but is still strongly related to PB1 and LBL3 phages (> 90%). In accordance with other related phages such as PB1 (Ceyssens *et al*., 2009), RNA polymerases are absent in all three phages, which is commonly observed for these types of phages, where transcription relies entirely on the host machinery. All three phages carry a DNA polymerase III alpha‐subunit that catalyses the polymerization reaction of the host DNA polymerase III holoenzyme, which is the DNA polymerase observed within bacteria (Kelman and O'Donnell, 1995). The presence of phage‐encoded DNA primase and helicase suggests that viral dsDNA elongation is performed by the phage independently of the host replication machinery. For the three phages the majority of genes are found in the positive strand (57.8%, 59.6% and 57.6%, for DL52, DL60 and DL68 respectively), whereas the others are found in the negative strand (42.2%, 40.4% and 42.4%, for DL52, DL60 and DL68 respectively).

**Figure 3:**
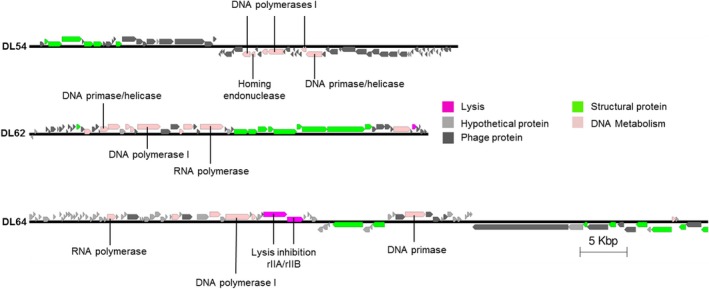
Representation of the annotated *P* *odoviridae* phages DL54, DLA62 and DL64. Nucleotide sequences were analysed using the Artemis tool and predicted reading frames are denoted by arrows.

The genome size observed for the *Podoviridae* phages DL54 and DL62 was similar, comprising 45 673 bp and 42 508 bp respectively. Phage DL64 genome was much larger comprising 72 378 bp (Fig. 3). A comparison between the nucleotide sequence of DL54, DL62 and DL64 showed no shared similarities. DL54 shares strong similarities (> 98%) with other lytic *Podoviridae* infecting *P. aeruginosa*, such as phiIBB‐PAA2 and LUZ24 (Ceyssens *et al*., 2008; Pires *et al*., 2014). Phage LUZ24 has two tRNA genes present in its genome; however, for DL54 tRNA was not observed in the genome sequence (Ceyssens *et al*., 2008). It is possible to recognize a conserved coding region responsible for phage assembly (large subunit of the terminase and upstream of the portal, capsid and scaffolding protein genes) and a region for DNA metabolism similarly, where the DNA polymerase was fragmented into three parts by an endonuclease gene, as observed for phage LUZ24 (Ceyssens *et al*., 2008). The DNA primase/helicase gene can also be found in the middle of the DNA polymerase. The two main modules are separated by several potential coding genes for phage proteins with unknown roles. The majority of genes, 50 (70.4%), are found in the positive strand, and 21 (29.6%) are found in the negative strand. The search using the blastn algorithm in the NCBI database showed that phage DL62 is closely related to phiKMV‐like phages (> 95%), such as phiKMV and LUZ19 (Lavigne *et al*., 2003; Lammens *et al*., 2009) and these phages are grouped into the T7‐like phage genus in the subfamily of the *Autographivirinae*. Genomes of T7‐type phage have been previously sequenced for phages of *Klebsiella pneumonia*, for example (Drulis‐Kawa *et al*., 2011), where genes are arranged into three clusters: early genes (cluster I), DNA metabolism (class II) and structural proteins for phage assembly and for host lysis (class III) (Lavigne *et al*., 2003). Such genome organization is clearly observed for phage DL62. However, differences can be found, such as the RNA polymerase that in T7‐like phages is present in cluster I of early genes and both in DL62 and phiKMV phages is present within the class II cluster. Typically the genome has a gene pairing lysin–holin in order to breakdown the bacterial cell. Phage DL62 carries a lysin (ORF51) found within cluster III, possibly with muraminidase activity, and the position and small size (66 residues) of ORF52 might code for the holin (Young and Bläsi, 1995; Wang *et al*., 2000). Like phiKMV (Lavigne *et al*., 2003), phage DL62 has a higher G + C% content (62.2%) when in comparison with the other phages of the cocktail, being close to the G + C% content of the bacterial host (65%) (Stover *et al*., 2000) and in accordance with the homologue phiKMV phage (Lavigne *et al*., 2003). Genes were all found in the forward strand, but the first gene is located in the reverse strand. Lastly, phage DL64 showed a unique genome sequence. The most striking feature of the genome is the presence of two genes (ORF41 and ORF42) homologous to rIIA‐like and rIIB‐like proteins from phage N4 that, although not yet clear, might play a role in the phenomenon of lysis inhibition and in other functions, such as interference in cell metabolism (Paddison *et al*., 1998). Lysis inhibition is defined as a delay of the phage in producing the enzyme holin while phage particles are being produced, resulting in their accumulation inside the host. Phage DL64 genome can be grouped in the N4‐like phages, such as LIT1 (98% nucleotide identity) and LUZ7 (73% nucleotide identity) that have been previously sequenced (Ceyssens *et al*., 2010) and several others on which genome description can be found in GenBank. N4‐like phages use three distinct RNA polymerases during their lytic infection cycle: a giant virion‐encapsulated RNA polymerase that is co‐injected with the phage genome into the host upon infection, a heterodimeric phage RNA polymerase transcribed from the middle region and a DNA‐binding protein that is able to arrest the host RNA polymerase to transcribe late phage proteins (Miller *et al*., 1997). Although, the blast search only predicted one RNA polymerase, we can presume that ORF15 might code for the giant RNA polymerase, as it is positioned in the early gene regions and its large size (3398 residues). It is suggested that the DNA‐binding protein coding gene might be ORF66 positioned directly upstream of the predicted RNA polymerase 1 (ORF67) with 88 residues. N4 phage codes for three tRNAs; however, no tRNAs were predicted for phage DL64 and phages LUZ7 and LIT1 (Ceyssens *et al*., 2010). No potential coding gene was predicted not only for the lytic enzyme for DL64, but also for LUZ7 and LIT1, and it is suggested that these phages might code for a novel type of endolysin or execute a different lysis mechanism (Ceyssens *et al*., 2010). A DNA polymerase I, a DNA helicase and primase are included in the genome of phage DL64, suggesting a host independency for DNA replication. The genome organization seems to be divided into two large clusters positioned on different strands and in their middle two small clusters are present, which are transcribed from opposite directions. This can also be observed for phages LUZ7 and LIT1 (Ceyssens *et al*., 2010). Hence, the first larger cluster includes transcription, DNA replication and lysis inhibition, a first small cluster that comprises a DNA primase, a second small cluster for structural proteins followed by the second large cluster for DNA package. The great majority of the genes, 70 (77.8%), are found in the leftward strand, and the other 20 genes (22.2%) in the opposite strand. The gene‐coding potential of these phages ranges from 1.243 to 1.391 genes per kilobase. Phage DL54 stands out from the other phages exhibiting the highest gene density of 1.554 genes per kilobase. Although for the great majority of potential coding regions (more than 60% for all cases) no known function has yet been found. The six DNA sequences were searched for the presence of antimicrobial genes and for any known virulence factor. Results from this search revealed that all six phages do not carry any potential genes coding for known antibiotic resistance or virulence factors and lysogeny modules were not found to be present. Table 3 summarizes the key features of the six genomes (see also Tables S1–S6 for general features of putative ORFs in the Supporting information).

**Table 3:** Summary of general genome features of the phage cocktail

Phage	Taxonomic family	Genome size	Number of predicted ORFs	G + C%	Gene density (genes per kilobase)	Coding%
DL52	*Myoviridae*	65 867	90	54.9	1.366	89.7
DL54	*Podoviridae*	45 673	71	52.4	1.554	92.4
DL60	*Myoviridae*	66 103	89	54.9	1.346	89.8
DL62	*Podoviridae*	42 508	55	62.2	1.293	94.4
DL64	*Podoviridae*	72 378	90	55.0	1.243	92.5
DL68	*Myoviridae*	66 111	92	55.7	1.391	91.6

### Bacteriophage mixture inhibits planktonic bacterial growth

**Figure 4:**
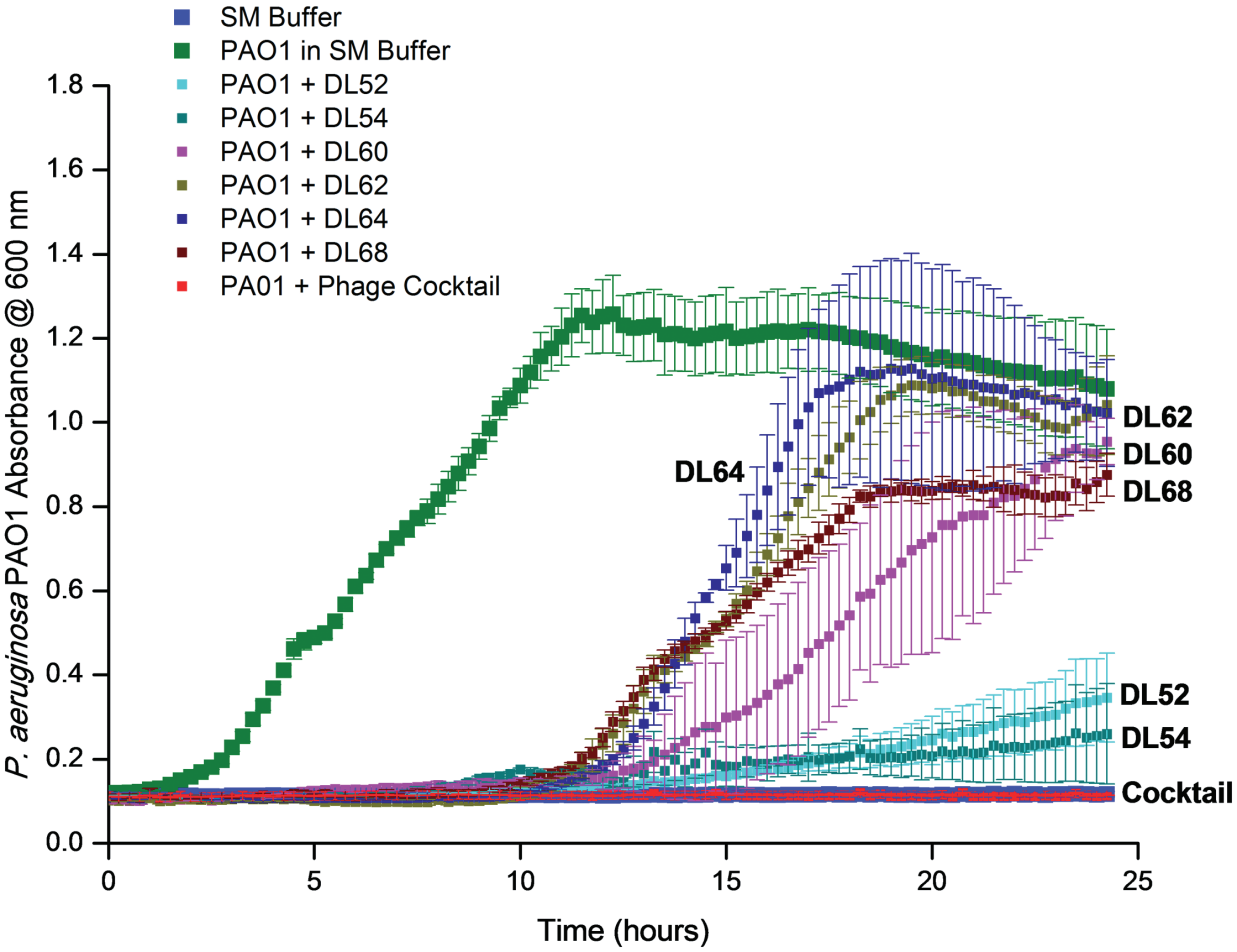
Dynamic of *P* *. aeruginosa* PAO1 bacteria with single phage and phage cocktail in liquid cultures over 24 h of incubation at 37°C. Absorbance readings at 600 nm were taken in a microtitre plate reader. *P* *seudomonas aeruginosa* PAO1 isolate growing with only SM buffer (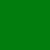), with single DL52 (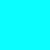), DL54 (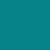), DL60 (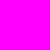), DL62 (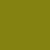), DL64 (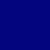) DL68 (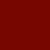) and with the phage cocktail in SM buffer (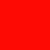) and also a negative‐control SM‐only buffer (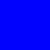) are shown in the figure. Assays were performed three times, and OD_600_ was expressed as the mean ± standard deviation.

The efficacy of each of the six individual phages and their combination into a cocktail was assessed by inoculating a bacterial broth culture of *P. aeruginosa* PAO1. Suspensions containing the phages were prepared to achieve a Multiplicity of Infectivity (MOI, the ratio of virus particles to viable bacterial cells) of 0.1 in the culture and this was introduced after 2 h of incubation of the latter. Growth continued for a further 22 h period. Figure 4 shows that for all individual phage suspensions there was eventually a regrowth of the PAO1 culture between 8 and 13 h after phage inoculation (time points: 10–15 h). Conversely, by using a suspension of the six phages in a cocktail, no regrowth and a full inhibition of the bacterial culture were observed.

### Bacteriophage mixture eradicates biofilms under static conditions

**Figure 5:**
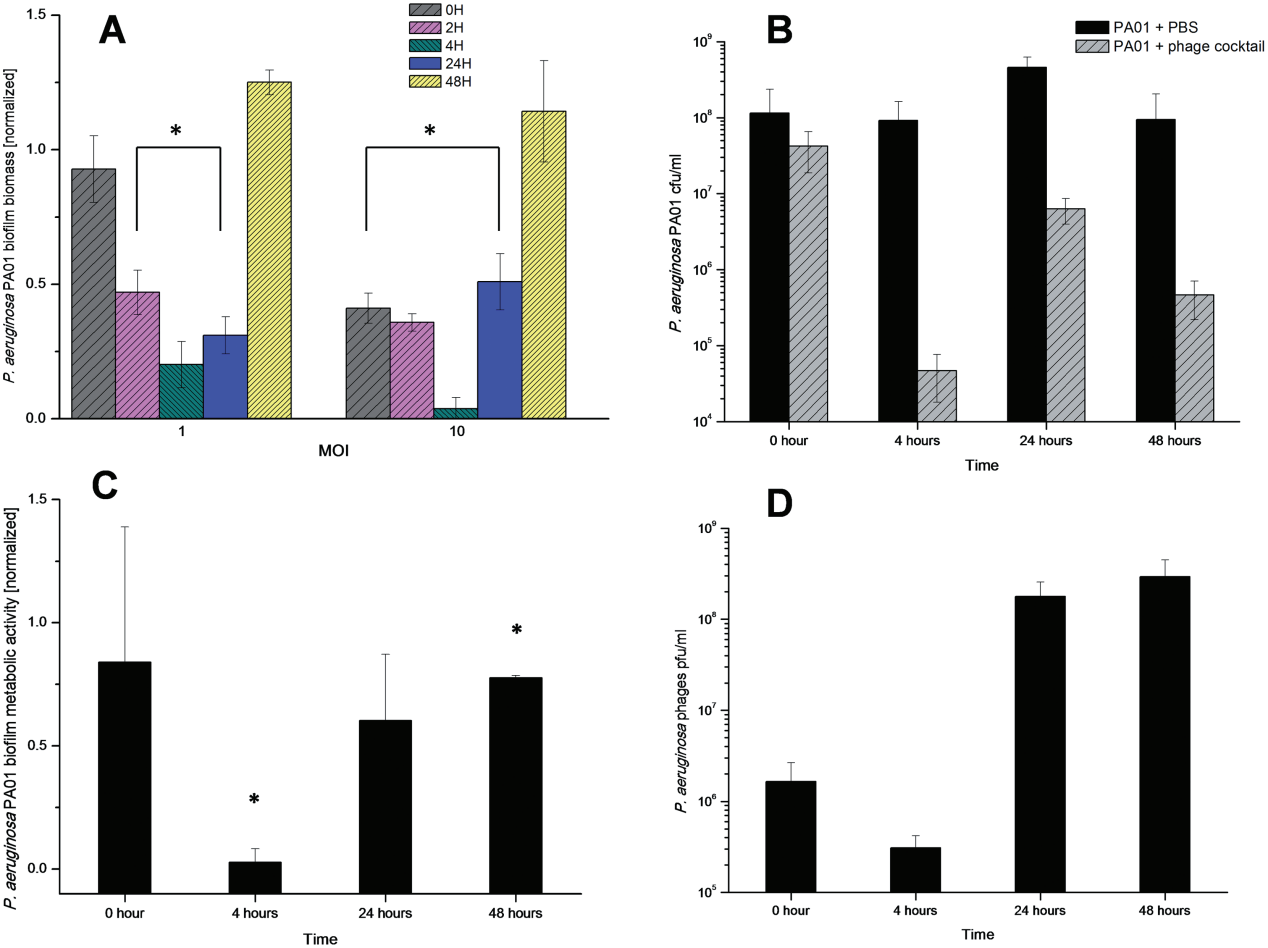
*P* *seudomonas aeruginosa* PAO1 biofilm treated with the phage cocktail over 48 h. (A) Normalized biofilm biomass treated with the phage cocktail at two different MOIs (OD_600_ reading after CV staining). (C) Normalized cell metabolic activity in the biofilm treated with the phage cocktail at MOI 1 (OD_600_ reading after XTT assay). (B) and (D) show the estimation of numbers of viable bacterial cells and phage particles in the biofilms treated with the phage cocktail. Assays were performed three times, and the means ± standard deviations are indicated. Statistical significance of biofilm reduction was assessed by performing Student's *t‐*test. *P* *‐*values are indicated (*P < 0.05).

In order to assess the potential of the phage cocktail to eradicate biofilms under static conditions, 48 h biofilms produced by *P. aeruginosa* PAO1 were established in 96‐well polystyrene microtitre plates. Washing steps were performed carefully as the mechanical wash step could disrupt the very viscous biofilms because of their high polysaccharides content. Established biofilms were treated with the phage cocktail at two different MOIs, 1 and 10, and their biomass density was assessed at 0, 2, 4, 24 and 48 h, by crystal violet staining followed by OD_600_ measurements (Fig. 5A). An 80% reduction of the biofilm biomass was observed in the first 4 h of phage treatment for MOI 1, and more than 95% reduction was observed for MOI 10 (*P* < 0.05) when compared with the PBS control. At 24 h, although biofilm biomass was still lower than for the PBS controls biofilm wells for both MOI cases (MOI of 10, *P* = 0.015; MOI of 1, *P* = 0.003) (Figs 5A and S7), there was biofilm regrowth compared with 4 h after phage addition. By the end of the experiment at 48 h, the biofilm biomass measured was greater than the biomass of the control biofilms. In parallel, an tetrazolium dye (XTT) assay was performed to evaluate the metabolic activity of the cells in the biofilm when treated with a phage cocktail suspension at MOI 10 over 48 h. In comparison with the PBS control a decrease in metabolic activity during the first 4 h of treatment (*P* < 0.05) was observed (Figs 5C and S7), and this was followed by a regrowth. Although in this case, by 48 h the regrown biofilm did not surpass the biomass of the biofilms inoculated only with PBS, showing lower numbers (*P* < 0.05). In a similar experiment, biofilms were disrupted and both bacterial and phages number were estimated. The same behaviour was observed, where in the first 4 h of treatment there was a clear reduction of the viable cell number, followed by a regrowth measured at 24 h. By 48 h a ∼ 1 log cell number reduction was observed; however, this was also seen with the disrupted control biofilm (Fig. 5B). Regarding the phage numbers in the biofilm, it was seen that at 4 h there was a decrease in phage concentration, most likely due to many of the phages still infecting the cells and not being present in the surrounding environment. At 24 and 48 h, the number of phages present in the biofilm increased by ∼ 2 logs (Fig. 5D). For all cases a higher MOI was found to be more effective in rapidly reducing the biofilm biomass and also better at preventing the consequent biofilm regrowth.

### Bacteriophage mixture eradicates biofilms under dynamic conditions

**Figure 6:**
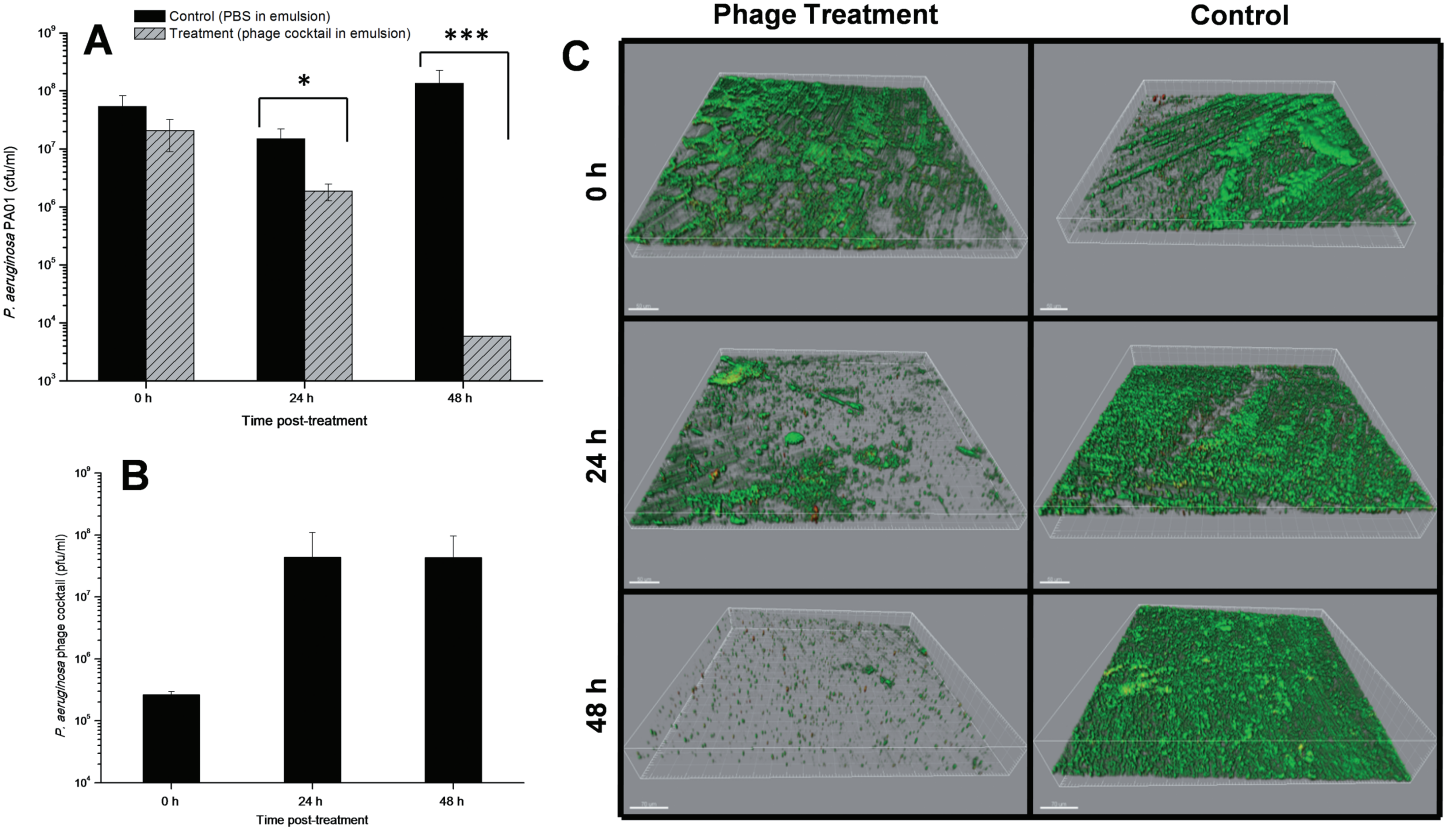
*P* *seudomonas aeruginosa* PAO1 48 h biofilm grown under flow conditions in the Robbins device and treated with a phage in emulsion suspension or PBS in emulsion suspension (control) for 48 h. (A) Number of viable cells recovered from the disruption of the biofilms; (B) number of phage particles recovered from the disputed biofilms; (C) 3D images of the biofilms observed under the confocal microscope stained with LIVE/DEAD stain at a magnification of 20×. Live (green) and dead (red) bacterial cells can be observed on the surface of the stainless steel disc. Enumeration assays were performed three times, and the means ± standard deviations are indicated. Statistical significance of biofilm reduction was assessed by performing Student's *t‐*test. *P* *‐*values are indicated (*P < 0.05; ***P < 0.001).

In order to mimic a more realistic infection situation a continuous growing culture of *P. aeruginosa* PAO1 was established to allow the production of a 48 h biofilm under dynamic flowing conditions. The biofilm was then treated with an emulsion suspension of the phage cocktail at an MOI of 0.1 and the biofilm was assessed for eradication and dispersion. Results showed a clear reduction of the biofilm and by 24 h of phage treatment this reduction was significantly different from the PBS‐treated biofilm control (Fig. 6A, *P* = 0.003) and by 48 h such reduction was even more evident (*P* = 0.00008). Via confocal microscopy, biofilms produced on the modified Robbins device (MRD) flow system were shown to have structures composed of a majority of live cells, although dead cells and possibly water channels could also be observed. Biofilm reduction and dispersion was also seen (Fig. 6C), and by 48 h the presence of a biofilm on the stainless steel discs was barely visible with only a few dispersed bacterial aggregates. These results are reinforced by an increase in the number of phages present in the disrupted biofilms of ∼ 3 logs (Fig. 6B). Also, the initial concentration of phage particles recovered from the disrupted biofilms was lower than the amount injected – indicating either washout of phage, or more likely lack of opportunity for infection and reproduction due to loss of biofilm.

## Discussion

In this study six phages were isolated from environmental sources and combined in a cocktail suspension that proved to be strongly lytic against a wide range of *P. aeruginosa* diverse clinical isolates, showing host coverage of 90% of the isolates collection tested. Based on transmission electron microscope (TEM) and genome sequencing, phages DL52, DL60 and DL68 belong to the *Myoviridae* long‐contractile tailed phage family and DL54, DL62 and DL64 belong to the *Podoviridae* short‐tailed phages family. Phage aggregation could be observed for phages 54 and 60, this behaviour is observed in nature (Hejkal *et al*., 1981; Narang and Codd, 1981) and has been attributed to pH, composition of ions and ionic strength. At the genome level all phages were revealed to be double‐stranded DNA (dsDNA), with high gene density and low G + C% compared with the host *P. aeruginosa* PAO1. The three *Myoviridae* phages were shown to be strongly related and they can be placed in the PB1‐like lytic virus group. It has been documented that these phages use the bacterial lipopolysaccharide as a receptor to attach to the cell (Jarrell and Kropinski, 1977) and we can assume that the same attachment mechanism might be employed by these three phages. Regarding the *Podoviridae* phages no interrelatedness was observed. Phage DL64 have a large genome compared with the other two, and DL54 showed the highest gene density. Interestingly, phage DL64 appears to carry the rIIA/rIIB lysis inhibition cassette, which is widely distributed among the genomes of phages infecting different host genera (Kushkina *et al*., 2013; Wittmann *et al*., 2014). This enables a delay in host lysis, but consequently results in a high increase in the burst size of the phage, providing phages of this type with a competitive advantage over others. If this type of phage is integrated within a therapeutic cocktail product it could be important to perform propagation separately; otherwise the phage might dominate the phage population. However, the role of such a cassette remains uncertain and several additional roles have been attributed to it, such as being involved in the host resistance mechanism of the abortive infection system (Labrie *et al*., 2010). No virulence or antibiotic resistance factors were identified in the genome, according to the data available at the moment, suggesting that the *P. aeruginosa* phage preparation could be safely used to treat infections related to these bacteria. However, the majority of putative coding regions of our phages do not have attributed function in common with the majority of sequenced phages in GenBank. Consequently, and in view of the potential of phage therapy as a reliable and safe therapeutic opportunity, it is important to further investigate the phage genomes in order to understand the function of gene products, as they can be involved in undesirable phage–host interaction or code for novel virulence factors (Lima‐Mendez *et al*., 2011).

Bacterial broth cultures growing with the phage cocktails showed an elimination of bacterial cell growth and suppression of resistant mutants of *P. aeruginosa* PAO1. Several studies have demonstrated that the use of a phage cocktail brings not only advantages regarding the target host range, but also in terms of preventing or delaying mutations in the population that can result in phage‐resistant clones and therapeutic failure (Tanji *et al*., 2004; Gu *et al*., 2012).

The great majority of microbial infections involve biofilms (James *et al*., 2008) and although assessment of phage lytic activity on bacterial planktonic growth is useful it is important that biofilm studies are performed when testing a new antibacterial therapeutic. Previous studies on *P. aeruginosa* suggest the potential utility of phages to reduce and/or eliminate biofilms produced by strains including PAO1 (Pires *et al*., 2011), and to prevent biofilm formation on indwelling medical devices, such as catheters (Fu *et al*., 2010). The first biofilm model system was performed under static conditions on a polystyrene surface for 48 h and phages were added to the biofilms. Biofilm biomass disruption was quickly observed after addition of the phage cocktail during the first 6 h of treatment. After 24 h, although the biofilm biomass was still lower than the PBS‐treated biofilm, it was clearly higher than the previous end‐point. By 48 h the biofilm showed a complete regrowth with a biomass higher than the non‐treated biofilms. The regrowth was not caused by a lack of phage particles because phages were recovered from the disrupted biofilms. This has also been observed elsewhere (Pires *et al*., 2011) and it might be a consequence of a longer interaction of the phage with the bacteria, also enhanced by a slower metabolic activity of the bacteria in the biofilm where phage proliferation is slowed down, resulting in the emergence of phage‐resistant clones in the bacterial population (Bohannan and Lenski, 2000). Comparing the effect of the phage cocktails on broth cultures to biofilms, a more positive and rapid effect was observed in eliminating the bacterial load of broth cultures than for biofilms, which were disrupted at a lower rate. This scenario is thought to be due to the metabolic state of the cells in a biofilm (Chen *et al*., 2012). This has been also been shown for antibiotic drugs where bacteria in biofilms can be much more resilient than those in planktonic culture (Hoyle and Costerton, 1991). A bacterial culture growing in a chemostat leads to constant concentrations of bacterial cells, their products, waste and media components and, hence, provides a controlled environment for bacteria to grow at a specific growth rate that will directly impact cell wall protein expression and secretion of extracellular polysaccharides (Terry *et al*., 1991). This in turn will ultimately determine how mature and protected the biofilm, which results from such cells, will be (McEldowney and Fletcher, 1986). The time taken for biofilm disruption in this system was longer, compared with that of the static biofilm model, and regrowth was not observed within the timeframe of the experiment. This might be related to the structure of the biofilms produced from a continuous culture and matured under flow conditions, which are likely to resemble more a natural biofilm.

The work presented here shows the characterization of six isolated phages for *P. aeruginosa* and assessment of their activity in different bacterial growth models: planktonic, static biofilm and dynamic biofilm. It highlights the importance of testing phage cocktails on different biofilm models, as expression of polysaccharides, cell signalling and ultimately resilience to external stresses is strongly affected by a myriad of factors and strategies, where the success of a therapeutic might be strongly dependent. The studies performed suggest that the use of phage cocktails, in this case against *P. aeruginosa*, could provide practical alternatives to antibiotic treatments for combating biofilm‐related infections and in particular the devastating effects of biofilm‐related CF infections; however, characterization and exploration of the cocktail biofilm eradication potential need to be further investigated.

## Experimental procedures

### Bacteria and growth conditions

*Pseudomonas aeruginosa* strains (listed in Table 1) used in this study include PAO1 and clinical isolates from acute and chronic infections, such as a bacteraemia and CF sputum. Bacteria from Luria–Bertani (LB) agar plates were grown at 37°C with constant shaking (170 rpm) in LB. LB agar and LB‐soft agar containing 0.65% bacteriological agar were used for bacteriophage propagation and plaque count assays. Note that the medium was supplemented with 1 mM CaCl_2_ and 1 mM MgSO_4_ to improve phage adsorption (Tucker, 1961). Bacterial aliquots were stocked at −80°C in broth containing 15% (v/v) glycerol.

### Bacteriophage isolation

Bacteriophages were isolated from crude sewage (Wessex Water, Bristol, UK) and flood water (Bath, UK) samples. Bacterial enrichments with *P. aeruginosa* isolates were performed to increase phage numbers as follows: 5 ml of actively growing *P. aeruginosa* cells (from overnight liquid culture in LB) and LB supplemented with 1 mM MgSO_4_ and 1 mM CaCl_2_ final concentration. The enrichment was incubated overnight at 37°C. A 10 ml of aliquot was taken from the overnight culture, and 1 M NaCl and 0.2% chloroform were added. The culture was then centrifuged (30 min, 3000 × *g*) to remove bacteria, and the supernatant was filtered‐sterilized (0.22 μm pore size; Millipore filter). This lysate (supernatant) was used to check the presence of lytic phages using the double layer method as described previously (Adams, 1959). Isolated single plaques were picked into SM buffer [100 mM NaCl, 8 mM MgSO4, 50 mM Tris‐HCl (pH 7.5), 0.01% (w/v) gelatine in MilliQ water (dH_2_O)], and successive rounds of single plaque purification were carried out until purified plaques were obtained, reflected by a single plaque morphology. Purified phages were stored in 50% (v/v) glycerol at −80°C for long‐term use. Short‐term stock preparations were maintained at 4°C.

### Bacteriophage propagation

Phage lysates were propagated on PAO1 strain. Briefly, 100 μl of phage lysate and 100 μl of host‐growing culture were mixed and left for 5 min at room temperature. Afterward, 3 ml of LB‐soft agar was added and poured onto LB agar plates. The following day, after an overnight incubation at 37°C, plates displaying confluent lysis were selected and 3 ml of SM buffer and 2% (v/v) chloroform were added before incubation at 37°C for 4 h. High‐titre phage solution was removed from the plates, centrifuged (8000 × *g*, 10 min) to remove cell debris, and then filter sterilized (pore size, 0.22 μm) and stored at 4°C.

### Sensitivity assay

To determine phage sensitivity of bacterial isolates, spot tests were performed. Briefly, 3 ml of LB‐soft agar was added to 100 μl of host‐growing culture and poured onto LB agar. Plates were left to dry for 20 min at 37°C. The different phage lysates were standardized to a titre of 10^9^ pfu/ml, and 10 μl of each lysate was spotted onto the bacterial lawns. This assay was performed in triplicate. The plates were allowed to dry before incubation overnight at 37°C. The following day, the sensitivity profiles of each of the bacterial strains were determined: if the bacterial lawn was lysed, slightly disrupted, or not disrupted, the bacterial isolate was labelled sensitive, intermediate or resistant to the phage infection respectively.

### Electron microscopy

Phage particles in water were deposited on carbon‐coated copper grids and negatively stained with 1% uranyl acetate (pH 4). Visualization was performed using a TEM (JEM1200EXII; JEOL, Bath, UK) operated at 120 kV. Size approximations were then performed using image analysis (ImageJ Software).

### Phage DNA extraction, DNA sequencing, analysis and assembly

Phage genomic DNA was extracted through a direct plaque sequencing described by Kot and colleagues (2014). DNA sequencing libraries were prepared using the Nextera XT DNA kit (Illumina, San Diego, CA, USA) according to the manufacturer's protocol. Individually tagged libraries were sequenced as a part of a flow cell as 2‐ by 250‐base paired‐end reads using the Illumina MiSeq platform (Illumina). Reads were analysed, trimmed and assembled using 6.5.1 CLC Genomic Workbench as described before (Kot *et al*., 2014). Genes were predicted and annotated using the rast server (Aziz *et al*., 2008). Phage genomes were searched for antimicrobial resistance determinants using the web‐tool resfinder (Zankari *et al*., 2012) that uses the blast algorithm (98% ID threshold). The web‐tool virulencefinder (Joensen *et al*., 2014) was used for the search of genes potential coding for virulence factors (98% ID threshold) found in *Escherichia coli*, *Enterococcus* spp and *Staphylococcus aureus*.

### Planktonic cultures treated with phage mixture

Planktonic cultures were performed in 96‐well microtitre plates, and their optical density was measured. In summary, a 1:100 dilution was prepared in the wells of the microplate by adding 5 μl of an overnight culture to 195 μl of LB. After 2 h of incubation at 37°C, the single or mixed phages at a desired MOI were added and the microplates were incubated for a further 22 h. The incubation was followed on a plate reader (FLUOstar Omega; BMG LabTech, UK) where the growth curves were established at an optical density at 600 nm (OD_600_). This approach allowed observation of the phage–bacterium interaction over time and also allows for monitoring of the appearance of resistant mutants for each phage lysate.

### Biofilm formation under static conditions

The biofilm assay for formation of *P. aeruginosa* biofilms was based on the previously described methods of Pires and colleagues (2011) with some modifications where appropriate. Formation of the biofilms was carried out in 96‐well polystyrene tissue culture microplates (Nunclon Delta Surface; Nunc, UK). An overnight culture was diluted to a titre of 10^8^ cfu/ml. In the microplate wells a 1:100 dilution was achieved by adding 5 μl of the bacterial suspension to 195 μl of half‐strength LB supplemented with 1% D‐(+)‐glucose (LBg) media, making a starting inoculum of 10^6^ cfu/ml. A total of 200 μl of broth only was added to a set of wells as a negative control. All wells were replicated three times. Afterwards, microplates were incubated at 37°C for 48 h with orbital constant shaking (120 rpm) for 48 h. Microplates were carefully wrapped with a layer of moistened blue paper towel roll and plastic to prevent the media in the microplates from evaporating. At 24 h, 50 μl of media was carefully removed from all wells and replenished with 50 μl of fresh 50% LB. Following incubation, media was poured off and wells were carefully washed twice with PBS to remove any planktonic cells. Microplates were allowed to dry for 1 h at 50°C. To determine total biofilm biomass, microplate wells were stained with 0.1% crystal violet (CV). After staining, the wells were washed twice with PBS solution and dried. Biofilm formation was determined by visual comparison of the stained wells and photographed.

### Biofilm treatment with phage mixture and analysis

Once biofilms were established and washed once with PBS, 100 μl of phage mixture in LB was added to a set of wells. Two MOIs were set up for the phage cocktail. A total of 100 μl of LB were added to both the positive and negative controls. All the experiments were performed three times. After static incubation at 37°C, microplates were washed and stained with crystal violet to determine biofilm biomass at predetermined time points. Optical density readings of the crystal violet staining intensity were performed: 100 μl of 95% (v/v) ethanol was added to each well and optical density at 600 nm (OD_600_) was measured using a plate reader. To determine the cellular activity of the biofilm an XTT reduction assay was performed after biofilms had been washed twice with PBS. Following this 1 ml of XTT solution was added to each well and the microtitre plates were then incubated in the dark for 3 h at 37°C with constant shaking (120 rpm). Absorbance was then measured using the FLUOstar plate reader at 490 nm. To determine the number of viable cells and phages particles within the biofilm, wells were washed twice with PBS and after 200 μl of PBS were added to the wells and the bottom and walls of the wells were scraped with a loop. The contents of each well were recovered with a pipette after five times pipetting up and down for complete disruption and detachment of cells to the surface. Serial dilutions were performed in ferrous ammonium sulfate (FAS), a virucide (McNerney *et al*., 1998) that ensures the inactivation of free phages prior to viable bacterial counts. A FAS stock solution was prepared immediately before use in 10 ml distilled water and filter sterilized (0.22 μm pore size) and was used at a working concentration of 10 mM. After standing at room temperature for 15 min, dilutions were plated out and bacterial colonies enumerated. For enumeration of phages present in the biofilm, an aliquot of 100 ml of the disrupted suspension was used to produce serial dilutions in PBS and phages were enumerated.

### Biofilm formation under flow conditions

A chemostat system was used to generate a continuous bacterial pure culture of *P. aeruginosa* PAO1 in order to provide inoculum to use in the MRD as described by Jass and collegueas (1995) with some modifications. The culture was grown in a 500 ml double wall chemostat vessel (Tyler Research Corporation, Canada) with constant stirring at 300 rpm at controlled temperature (37°C ± 0.2). A 10 ml sample of an overnight culture with 10^8^ cfu/ml was used to inoculate 500 ml of sterile fresh LB. Following incubation, the stirred chemostat was operated as a batch culture for 4 h to allow the establishment of the culture. After, the medium volume inside the vessel was kept at 500 ml and perfused with sterile LB at a dilution rate of 0.1 h^−1^. The chemostat was run over 48 h for the culture to achieve the steady state, with a constant population size of 3.6 × 10^7^ cfu/ml being established. A stainless steel Robbins Device (Tyler Research Corporation, Canada) was used to establish a biofilm. The device provides quantifiable samples of biofilm that can be monitored over time. It consists of hollow rectangular stainless steel tubes with 12 evenly spaced sampling ports. Each port allowed the insertion of a sampling stud that contained a stainless steel disc that lay flush to the inner surface of the lumen being subjected to the flow media and bacteria. Every stud was dipped into a sterile solution of mucin (100 μg/ml) to improve attachment of bacterial cells (McGuckin *et al*., 2011). The MRD was fitted into a thermal liquid regulated container (Tyler Research Corporation) in order to maintain the internal lumen temperature at 37°C. The culture growing under steady state was pumped through the MRD over 24 h, via the effluent tubing (flow rate = 250 μl/min) of the chemostat and biofilms were allowed to develop. Over this period the culture feed was replaced by fresh half‐strength media supplemented with 0.1% D‐(+)‐glucose (LBg) for another 24 h. At this time point, biofilms had been growing for 48 h, and fresh media feeding was paused in order to inoculate into the system 10 ml of 10^7^ pfu/ml phage in an oil‐in‐water nano‐emulsion suspension (organic phase: 5% (w/w) soybean oil; surfactant: 15% (w/w) Brij O10; aqueous phase: SM Buffer) performed through thermal phase inversion emulsification according to the method described previously (Esteban *et al*., 2014) and the control. Such emulsion was used to promote the lytic activity of the phage particles, as they provide a more stable environment for adsorption to the cells to occur (Esteban *et al*., 2014). Once the suspension was inside the MRD the inlet and outlet tubing of the MRD were clamped for a period of 20 min and phages were allowed to adsorb to the bacteria in the biofilms. One hour later the inlet and outlet tubing of the MRD were clamped again, and four random studs were removed in order to sample the biofilm communities formed. The removed studs were replaced with new sterile studs. The stainless steel discs were unscrewed from the studs and then three were processed for viable bacterial and phage counts and the fourth was prepared for analysis by the confocal laser scanning microscope (CLSM). Studs were removed from the MRD at predetermined time intervals: 0, 24 and 48 h.

### Viable bacterial and phage counts on MRD disks

Disks were carefully detached using tweezers from the stud and rinsed twice by dipping the disk in 1 ml in of PBS to remove planktonic and loosely adherent cells. The disk was placed in 1 ml PBS contained in 1 ml centrifuge tube and sonicated for six min (60W) before vortexing for 2 min to ensure complete detachment of the biofilm from the disk. For enumeration of viable bacterial cells, serial dilutions were performed in FAS virucide, and numbers determined by standard plate assay. For enumeration of phages present in the biofilm, 100 μl of the disrupted biofilm suspension was serially diluted and titrations were performed for phage enumeration. All the manipulation was performed inside a filtered lamina flow cabinet to prevent introduction of contamination.

### CLSM analysis on MRD disks

Discs were carefully removed and the biofilm surface was washed twice with PBS. Biofilm cell viability was determined using LIVE/DEAD® BacLight™ Bacterial Viability Kit staining following the manufacturer's instructions. The discs were immersed in the staining solution and incubated at room temperature for 15 min in the dark. After staining, the discs were gently rinsed with PBS and this was repeated six times more. The biofilm images were acquired in a Zeiss LSM510META CLSM. Biofilms were observed using the 20× objective (20×/0.5 W). Images were acquired with 1024 × 1024 resolutions in at least three different regions of the biofilm surface. Manipulation of the images and 3D reconstructions were performed using LSM Image Browser and imaris 7.4.2 software.

### Data analysis

Comparisons between the different time points and the positive controls were made by performing Student's *t*‐test, with a *P*‐value of 0.05 or less was considered statistically significant for all cases. All tests were performed with a confidence level of 95%. Spread of data at the 95% CI was estimated using the winpepi freeware statistical analysis program (Abramson, 2011).

### Nucleotide sequence accession numbers

The genome sequences of the isolated phages DL52–DL68 have been deposited in the GenBank database under accession number KR054028‐KR054033.

## Conflict of interest

None declared.

## Supporting information


**Table S1.** General features of putative ORFs from phage DL52 with best matches in the NCBI database.
**Table S2.** General features of putative ORFs from phage DL54 with best matches in the NCBI database.
**Table S3.** General features of putative ORFs from phage DL60 with best matches in the NCBI database.
**Table S4.** General features of putative ORFs from phage DL62 with best matches in the NCBI database.
**Table S5.** General features of putative ORFs from phage DL64 with best matches in the NCBI database.
**Table S6.** General features of putative ORFs from phage DL68 with best matches in the NCBI database.
