# Supplementary material for: A novel bacteriophage cocktail reduces and disperses P seudomonas aeruginosa biofilms under static and flow conditions
Source: Microb Biotechnol. 2015 Sep 8;9(1):61–74. doi: 10.1111/1751-7915.12316 (PMC4720417; doi:10.1111/1751-7915.12316)
Supplement: Supplementary file 1 — Table S1. General features of putative ORFs from phage DL52 with best matches in the NCBI database. Table S2. General features of putative ORFs from phage DL54 with best matches in the NCBI database. Table S3. General features of putative ORFs from phage DL60 with best matches in the NCBI database. Table S4. General features of putative ORFs from phage DL62 with best matches in the NCBI database. Table S5. General features of putative ORFs from phage DL64 with best matches in the NCBI database. Table S6. General features of putative ORFs from phage DL68 with best matches in the NCBI database. [file MBT2-9-061-s001.docx]

**Supplemental Information**

**Table S1:** General features of putative ORFs from phage DL52 with best matches in the NCBI database.

| **ORF** | **bp** | | **Strand** | **Gene Length** | **Representative similarity to proteins in database** | **% Identity** | **E-value** | **Accession no.** |
| --- | --- | --- | --- | --- | --- | --- | --- | --- |
|  | **Start** | **Stop** |  |  |  |  |  |  |
| **1** | 65 | 466 | + | 402 | Phage protein | 96 | 8E-87 | YP_001294433 |
| **2** | 498 | 821 | + | 324 | Phage protein | 100 | 3E-74 | YP_001294432 |
| **3** | 818 | 1021 | + | 204 | Phage protein | 100 | 1E-30 | YP_009009036 |
| **4** | 1027 | 1338 | + | 312 | Phage protein | 96 | 7E-64 | YP_002154157 |
| **5** | 1587 | 1934 | + | 348 | Phage protein | 97 | 1E-74 | YP_001294430 |
| **6** | 2038 | 2970 | + | 933 | Phage protein | 99 | 0 | YP_002455942 |
| **7** | 3072 | 3659 | + | 588 | Phage protein | 99 | 3E-142 | YP_009009031 |
| **8** | 3677 | 4114 | + | 438 | Phage protein | 97 | 3E-81 | YP_001294427 |
| **9** | 4200 | 4979 | + | 780 | Phage protein | 100 | 0 | YP_001294426 |
| **10** | 4982 | 5383 | + | 402 | Phage protein | 90 | 9E-71 | YP_001294425 |
| **11** | 5428 | 5778 | + | 351 | Phage protein | 99 | 7E-66 | YP_001294424 |
| **12** | 5778 | 5996 | + | 219 | Phage protein | 100 | 5E-45 | YP_001294423 |
| **13** | 5993 | 6376 | + | 384 | Phage protein | 98 | 3E-65 | YP_001294422 |
| **14** | 6414 | 7796 | - | 1383 | Phage terminase, large subunit | 100 | 0 | YP_001294421 |
| **15** | 7998 | 8186 | + | 189 | Phage protein | 98 | 1E-22 | YP_001294420 |
| **16** | 8307 | 8453 | + | 147 | Phage protein | 100 | 9E-12 | YP_001294419 |
| **17** | 8465 | 8767 | + | 303 | Phage protein | 95 | 3E-60 | YP_001294418 |
| **18** | 8814 | 9731 | + | 918 | Phage tail length tape-measure protein | 99 | 6E-123 | YP_002456023 |
| **19** | 9734 | 9922 | + | 189 | Phage protein | 97 | 4E-38 | YP_001294508 |
| **20** | 10002 | 10187 | + | 186 | Phage protein | 98 | 3E-34 | YP_001294507 |
| **21** | 10312 | 10512 | + | 201 | Phage protein | 98 | 3E-39 | YP_001294505 |
| **22** | 10509 | 10724 | + | 216 | Phage protein | 99 | 8E-42 | YP_001294504 |
| **23** | 10721 | 10924 | + | 204 | Phage protein | 61 | 6E-18 | YP_001294503 |
| **24** | 10908 | 11120 | + | 213 | Phage protein | 90 | 1E-40 | YP_001294502 |
| **25** | 11149 | 11796 | + | 648 | Phage protein | 93 | 2E-135 | YP_001294501 |
| **26** | 11940 | 12098 | - | 159 | hypothetical protein | - | - | not available |
| **27** | 12185 | 12409 | + | 225 | Phage protein | 93 | 1E-42 | YP_002154315 |
| **28** | 12465 | 12692 | + | 228 | Phage protein | 75 | 4E-32 | YP_006200849 |
| **29** | 12702 | 12923 | + | 222 | Phage protein | 81 | 2E-33 | YP_007002628 |
| **30** | 12971 | 13288 | + | 318 | Phage protein | 93 | 5E-69 | YP_002456011 |
| **31** | 13298 | 13906 | + | 609 | Phage protein | 91 | 2E-106 | YP_002456010 |
| **32** | 14098 | 14712 | + | 615 | Phage protein | 99 | 7E-92 | YP_002154310 |
| **33** | 14880 | 15452 | - | 573 | Phage protein | 98 | 1E-119 | YP_001294493 |
| **34** | 16425 | 18164 | - | 1740 | DNA primase, phage associated | 99 | 0 | YP_002456007 |
| **35** | 18312 | 18497 | - | 186 | Phage protein | 100 | 6E-14 | YP_001294490 |
| **36** | 18503 | 19579 | - | 1077 | Phage protein | 99 | 0 | YP_002364381 |
| **37** | 19576 | 20025 | - | 450 | Phage protein | 97 | 2E-102 | YP_007238225 |
| **38** | 20025 | 20579 | - | 555 | Phage protein | 91 | 3E-29 | WP_023104124 |
| **39** | 20999 | 21784 | - | 786 | Phage protein | 99 | 1E-180 | YP_002456002 |
| **40** | 21952 | 22374 | + | 423 | Phage protein | 99 | 2E-96 | YP_002456001 |
| **41** | 22361 | 23548 | + | 1188 | DNA helicase, phage-associated | 99 | 0 | YP_002456000 |
| **42** | 23710 | 24600 | + | 891 | Phage protein | 100 | 2E-68 | YP_002154209 |
| **43** | 24705 | 25706 | + | 1002 | Phage protein | 99 | 0 | YP_002455998 |
| **44** | 25796 | 26026 | + | 231 | Phage protein | 97 | 1E-35 | YP_002455997 |
| **45** | 26026 | 26244 | + | 219 | Phage protein | 97 | 2E-30 | YP_002154206 |
| **46** | 26228 | 26446 | + | 219 | Phage tail assembly | 97 | 2E-44 | YP_001294479 |
| **47** | 26446 | 26706 | + | 261 | Phage protein | 94 | 2E-50 | YP_002455994 |
| **48** | 26718 | 26924 | + | 207 | Phage protein | 96 | 6E-39 | YP_001294477 |
| **49** | 26924 | 27841 | + | 918 | Thymidylate synthase thyX | 100 | 0 | YP_001294476 |
| **50** | 27843 | 28034 | + | 192 | Phage protein | 100 | 4E-35 | YP_001294475 |
| **51** | 28037 | 29077 | + | 1041 | 3'-phosphatase, 5'-polynucleotide kinase, phage-associated | 94 | 0E+00 | YP_002455989 |
| **52** | 29153 | 29707 | + | 555 | Phage protein | 99 | 1E-132 | YP_001294473 |
| **53** | 29707 | 32814 | + | 3108 | DNA polymerase III alpha subunit | 99 | 0 | YP_002455987 |
| **54** | 32807 | 33217 | + | 411 | Phage protein | 100 | 3E-81 | YP_001294471 |
| **55** | 33214 | 34773 | + | 1560 | DNA helicase, phage-associated | 99 | 0 | YP_001294470 |
| **56** | 34868 | 35488 | + | 621 | Phage protein | 100 | 3E-120 | YP_002154195 |
| **57** | 35577 | 36476 | + | 900 | Phage protein | 100 | 0 | YP_002154194 |
| **58** | 36533 | 37138 | + | 606 | Phage protein | 100 | 2E-100 | YP_001294467 |
| **59** | 37135 | 37689 | + | 555 | Phage DNA-binding protein | 98 | 1E-128 | YP_002154192 |
| **60** | 37744 | 38655 | + | 912 | DNA ligase, phage-associated | 96 | 0 | YP_002154191 |
| **61** | 38935 | 39186 | + | 252 | Phage protein | - | - | not available |
| **62** | 39210 | 39872 | - | 663 | Lytic enzyme | - | - | not available |
| **63** | 39872 | 40300 | - | 429 | Phage protein | - | - | not available |
| **64** | 40302 | 43196 | - | 2895 | Phage tail fibers | - | - | not available |
| **65** | 43201 | 44715 | - | 1515 | Phage protein | - | - | not available |
| **66** | 44712 | 45965 | - | 1254 | Phage protein | - | - | not available |
| **67** | 46022 | 46453 | - | 432 | Phage baseplate | - | - | not available |
| **68** | 46433 | 46666 | - | 234 | hypothetical protein | - | - | not available |
| **69** | 46743 | 47276 | - | 534 | Phage protein | - | - | not available |
| **70** | 47276 | 48139 | - | 864 | Phage protein | - | - | not available |
| **71** | 48139 | 50721 | - | 2583 | Phage internal (core) protein | - | - | not available |
| **72** | 50725 | 51153 | - | 429 | Phage protein | - | - | not available |
| **73** | 51163 | 51756 | - | 594 | Phage tail fiber protein | - | - | not available |
| **74** | 51765 | 52169 | - | 405 | Phage protein | - | - | not available |
| **75** | 52304 | 52807 | - | 504 | Phage protein | - | - | not available |
| **76** | 52817 | 53245 | - | 429 | Phage protein | - | - | not available |
| **77** | 53594 | 53917 | - | 324 | Phage protein | - | - | not available |
| **78** | 53917 | 54369 | - | 453 | Phage tail fiber protein | - | - | not available |
| **79** | 54427 | 55941 | - | 1515 | Phage protein | - | - | not available |
| **80** | 56536 | 57087 | - | 552 | Phage protein | - | - | not available |
| **81** | 57095 | 57493 | - | 399 | Phage protein | 95 | 2E-85 | YP_001294443 |
| **82** | 57490 | 57957 | - | 468 | Phage protein | 99 | 6E-76 | YP_001294442 |
| **83** | 57972 | 58409 | - | 438 | Phage protein | 100 | 4E-103 | YP_001294441 |
| **84** | 58511 | 59659 | - | 1149 | Phage capsid and scaffold | 99 | 0E+00 | YP_002455954 |
| **85** | 59669 | 60304 | - | 636 | Phage protein | 100 | 2E-145 | YP_001294439 |
| **86** | 60308 | 61744 | - | 1437 | Phage protein | 100 | 0E+00 | YP_001294438 |
| **87** | 62259 | 62396 | - | 138 | Phage protein | 98 | 3E-21 | YP_002455951 |
| **88** | 62393 | 62599 | - | 207 | Phage protein | 99 | 1E-39 | YP_001294436 |
| **89** | 62619 | 63455 | - | 837 | Phage minor capsid protein | 100 | 0 | YP_002455949 |
| **90** | 63455 | 65752 | - | 2298 | Phage-related protein | 99 | 0 | YP_001294434 |

**Table S2:** General features of putative ORFs from phage DL54 with best matches in the NCBI database.

| **ORF** | **bp** | | **Strand** | **Gene Length** | **Representative similarity to proteins in database** | **% Identity** | **E-value** | **Accession no.** |
| --- | --- | --- | --- | --- | --- | --- | --- | --- |
|  | **Start** | **Stop** |  |  |  |  |  |  |
| **1** | 1147 | 1605 | + | 459 | Phage protein | 99 | 1E-105 | YP_008857869 |
| **2** | 1637 | 2035 | + | 399 | Phage baseplate hub | 99 | 5E-90 | YP_001671940 |
| **3** | 2035 | 3483 | + | 1449 | Phage terminase, large subunit | 100 | 0 | YP_001671939 |
| **4** | 3483 | 5603 | + | 2121 | Phage portal (connector) protein | 100 | 0 | YP_008857866 |
| **5** | 5633 | 5848 | + | 216 | Phage protein | 100 | 3E-43 | YP_001671937 |
| **6** | 5848 | 6840 | + | 993 | Phage capsid and scaffold | 100 | 1E-164 | YP_009125634 |
| **7** | 6859 | 7812 | + | 954 | Phage capsid and scaffold | 100 | 0 | YP_001671935 |
| **8** | 7861 | 8181 | + | 321 | Phage protein | 100 | 3E-71 | YP_001671934 |
| **9** | 8185 | 8811 | + | 627 | Phage protein | 100 | 1E-139 | YP_001671933 |
| **10** | 8822 | 9013 | + | 192 | Phage protein | 100 | 4E-34 | YP_001671932 |
| **11** | 8997 | 9245 | + | 249 | Phage protein | 100 | 1E-27 | YP_001671931 |
| **12** | 9235 | 9882 | + | 648 | Phage tail fibers | 100 | 3E-152 | YP_001671930 |
| **13** | 9891 | 11432 | + | 1542 | Phage protein | 100 | 0 | AGC35290 |
| **14** | 11429 | 12118 | + | 690 | Phage protein | 100 | 2E-142 | YP_001671928 |
| **15** | 12115 | 12549 | + | 435 | Phage protein | 99 | 3E-102 | YP_001671927 |
| **16** | 12530 | 13471 | + | 942 | Phage protein | 99 | 2E-151 | YP_008857854 |
| **17** | 13601 | 13831 | + | 231 | hypothetical protein | 100 | 1E-43 | YP_009125623 |
| **18** | 13856 | 15370 | + | 1515 | Phage protein | 100 | 0 | YP_008857852 |
| **19** | 15521 | 18688 | + | 3168 | Phage protein | 100 | 0 | YP_001671923 |
| **20** | 18700 | 19587 | + | 888 | Phage protein | 95 | 0 | YP_008857850 |
| **21** | 19602 | 19958 | + | 357 | Phage protein | 97 | 2E-76 | YP_009007837 |
| **22** | 20165 | 20371 | - | 207 | Phage protein | 100 | 2E-22 | YP_001671920 |
| **23** | 20358 | 20567 | - | 210 | Phage protein | 100 | 3E-42 | YP_008857847 |
| **24** | 20571 | 20789 | - | 219 | Phage protein | 100 | 8E-44 | YP_001671918 |
| **25** | 20786 | 21547 | - | 762 | Phage protein | 100 | 0 | YP_001671917 |
| **26** | 21540 | 21779 | - | 240 | Phage protein | 99 | 2E-50 | YP_009125613 |
| **27** | 21733 | 22713 | - | 981 | Phage protein | 100 | 0 | YP_008857844 |
| **28** | 22694 | 23524 | - | 831 | DNA polymerase I | 99 | 0 | YP_009007830 |
| **29** | 23609 | 24058 | - | 450 | Phage-associated homing endonuclease | 100 | 7E-106 | YP_001671913 |
| **30** | 24058 | 24342 | - | 285 | Phage protein | 98 | 4E-58 | YP_009125609 |
| **31** | 24314 | 24724 | - | 411 | Phage protein | 99 | 4E-92 | YP_009007827 |
| **32** | 24795 | 25349 | - | 555 | Helix-destabilizing protein | 100 | 1E-115 | YP_001671910 |
| **33** | 25419 | 27056 | - | 1638 | DNA polymerase, phage-associated | 100 | 0 | YP_001671907 |
| **34** | 27057 | 27254 | - | 198 | Phage protein | 98 | 2E-37 | YP_008857837 |
| **35** | 27259 | 27768 | - | 510 | Phage protein | 86 | 2E-105 | YP_008857836 |
| **36** | 27845 | 28123 | - | 279 | Phage protein | 99 | 7E-57 | AGC35267 |
| **37** | 28114 | 28275 | - | 162 | Phage protein | 100 | 1E-28 | AGC35266 |
| **38** | 28265 | 28456 | - | 192 | Phage protein | 98 | 4E-37 | YP_001671901 |
| **39** | 28437 | 28670 | - | 234 | Phage protein | 100 | 2E-32 | YP_008857832 |
| **40** | 28706 | 29002 | - | 297 | Phage protein | 98 | 4E-56 | YP_009007816 |
| **41** | 28984 | 29493 | - | 510 | DNA polymerase, phage-associated | 99 | 2E-121 | YP_009007815 |
| **42** | 29477 | 31186 | - | 1710 | DNA primase/helicase, phage-associated | 100 | 0 | AGC35261 |
| **43** | 31187 | 31564 | - | 378 | Phage protein | 100 | 3E-87 | YP_008857828 |
| **44** | 31564 | 31962 | - | 399 | Phage protein | 100 | 2E-91 | YP_001671895 |
| **45** | 31962 | 32846 | - | 885 | Phage protein | 99 | 0 | YP_001671894 |
| **46** | 32977 | 33198 | - | 222 | Phage protein | 100 | 4E-44 | YP_001671893 |
| **47** | 33208 | 34740 | - | 1533 | Phage protein | 100 | 0 | YP_008857824 |
| **48** | 34752 | 35927 | - | 1176 | Phage protein | 99 | 0 | YP_008857823 |
| **49** | 35903 | 36472 | - | 570 | Phage protein | 100 | 6E-136 | YP_001671890 |
| **50** | 36465 | 37265 | - | 801 | Phage protein | 98 | 0 | YP_009125589 |
| **51** | 37262 | 38218 | - | 957 | Phage protein | 99 | 0 | YP_008857820 |
| **52** | 38237 | 39199 | - | 963 | Phage protein | 98 | 0 | YP_008857819 |
| **53** | 39270 | 39398 | - | 129 | hypothetical protein | 98 | 2E-20 | YP_009125586 |
| **54** | 39410 | 40234 | - | 825 | Phage protein | 100 | 1E-170 | YP_009007804 |
| **55** | 40244 | 40462 | - | 219 | hypothetical protein | 96 | 4E-43 | YP_009125584 |
| **56** | 40469 | 40687 | - | 219 | hypothetical protein | 49 | 4E-13 | YP_009125583 |
| **57** | 40689 | 40901 | - | 213 | Phage protein | 83 | 4E-32 | AGC35246 |
| **58** | 41032 | 41457 | - | 426 | Phage protein | 96 | 2E-95 | YP_001671885 |
| **59** | 41457 | 41708 | - | 252 | hypothetical protein | 60 | 9E-14 | AGC35244 |
| **60** | 41705 | 41875 | - | 171 | Phage protein | 100 | 3E-31 | AGC35243 |
| **61** | 41875 | 42141 | - | 267 | Phage protein | 97 | 2E-55 | YP_001671884 |
| **62** | 42146 | 42391 | - | 246 | Phage protein | 100 | 5E-52 | YP_008857811 |
| **63** | 42400 | 42546 | - | 147 | Phage protein | 100 | 4E-27 | NP_775198 |
| **64** | 42533 | 42688 | - | 156 | hypothetical protein | 100 | 2E-28 | YP_008857809 |
| **65** | 42699 | 42974 | - | 276 | Phage protein | 100 | 2E-59 | YP_008857808 |
| **66** | 42976 | 43209 | - | 234 | hypothetical protein | 100 | 1E-47 | YP_001671880 |
| **67** | 43341 | 43523 | - | 183 | Phage protein | 100 | 7E-33 | YP_001671879 |
| **68** | 43703 | 43843 | - | 141 | Phage protein | 100 | 8E-24 | NP_775190 |
| **69** | 43840 | 44028 | - | 189 | Phage protein | 98 | 9E-35 | YP_008857803 |
| **70** | 44131 | 44613 | - | 483 | Phage protein | 98 | 1E-107 | YP_008857802 |
| **71** | 44617 | 44922 | - | 306 | Phage protein | 100 | 7E-57 | YP_008857801 |

**Table S3:** General features of putative ORFs from phage DL60 with best matches in the NCBI database.

| **ORF** | **bp** | | **Strand** | **Gene Length** | **Representative similarity to proteins in database** | **% Identity** | **E-value** | **Accession no.** |
| --- | --- | --- | --- | --- | --- | --- | --- | --- |
|  | **Start** | **Stop** |  |  |  |  |  |  |
| **1** | 53 | 466 | + | 414 | Phage protein | 99 | 1E-91 | YP_002455947 |
| **2** | 498 | 821 | + | 324 | Phage protein | 98 | 9E-73 | YP_002455946 |
| **3** | 793 | 909 | + | 117 | Phage protein | 92 | 1E-14 | YP_001294431 |
| **4** | 1027 | 1338 | + | 312 | Phage protein | 100 | 3E-68 | YP_002455944 |
| **5** | 1587 | 1934 | + | 348 | Phage protein | 97 | 1E-74 | YP_002455943 |
| **6** | 2038 | 2967 | + | 930 | Phage protein | 95 | 0E+00 | YP_001294429 |
| **7** | 3069 | 3656 | + | 588 | Phage protein | 98 | 8E-140 | YP_009009031 |
| **8** | 3672 | 4109 | + | 438 | Phage protein | 89 | 7E-73 | YP_001294427 |
| **9** | 4195 | 4974 | + | 780 | Phage protein | 99 | 0 | YP_002154152 |
| **10** | 4977 | 5378 | + | 402 | Phage protein | 99 | 6E-61 | YP_001294425 |
| **11** | 5423 | 5773 | + | 351 | Phage protein | 98 | 9E-65 | YP_001294424 |
| **12** | 5773 | 5991 | + | 219 | Phage protein | 99 | 4E-44 | YP_001294423 |
| **13** | 5988 | 6371 | + | 384 | Phage protein | 98 | 2E-82 | YP_002455935 |
| **14** | 6408 | 7790 | - | 1383 | Phage terminase, large subunit | 100 | 0 | YP_001294421 |
| **15** | 7967 | 8113 | + | 147 | Phage protein | 100 | 9E-12 | YP_001294419 |
| **16** | 8126 | 8428 | + | 303 | Phage protein | 100 | 7E-63 | YP_001294418 |
| **17** | 8475 | 9392 | + | 918 | Phage tail length tape-measure protein | 99 | 2E-123 | YP_002456023 |
| **18** | 9395 | 9583 | + | 189 | Phage protein | 98 | 8E-38 | YP_001294508 |
| **19** | 9670 | 9921 | + | 252 | Phage protein | 94 | 5E-50 | YP_002154231 |
| **20** | 9918 | 10118 | + | 201 | Phage protein | 97 | 7E-38 | YP_001294505 |
| **21** | 10115 | 10330 | + | 216 | Phage protein | 100 | 2E-42 | YP_001294504 |
| **22** | 10339 | 10518 | + | 180 | Phage protein | 98 | 2E-33 | YP_001294503 |
| **23** | 10515 | 10727 | + | 213 | Phage protein | 96 | 1E-42 | YP_001294502 |
| **24** | 10755 | 11402 | + | 648 | Phage protein | 99 | 1E-143 | YP_001294501 |
| **25** | 11399 | 11725 | + | 327 | Phage protein | 99 | 4E-72 | YP_002154225 |
| **26** | 11791 | 12015 | + | 225 | Phage protein | 93 | 1E-42 | YP_002154315 |
| **27** | 12069 | 12296 | + | 228 | Phage protein | 68 | 1E-25 | WP_041598427 |
| **28** | 12306 | 12527 | + | 222 | Phage protein | 85 | 9E-36 | YP_007002628 |
| **29** | 12575 | 12892 | + | 318 | Phage protein | 93 | 8E-69 | YP_002456011 |
| **30** | 12902 | 13507 | + | 606 | Phage protein | 98 | 7E-116 | YP_002456010 |
| **31** | 13700 | 14314 | + | 615 | Phage protein | 99 | 1E-93 | YP_002154219 |
| **32** | 14485 | 15057 | - | 573 | Phage protein | 99 | 3E-120 | YP_001294493 |
| **33** | 16029 | 17759 | - | 1731 | DNA primase, phage associated | 99 | 0 | YP_001294491 |
| **34** | 17907 | 18092 | - | 186 | Phage protein | 93 | 7E-06 | YP_001294490 |
| **35** | 19171 | 19620 | - | 450 | Phage protein | 99 | 6E-103 | YP_007002619 |
| **36** | 19644 | 21194 | - | 1551 | Phage protein | 93 | 8E-157 | YP_006200837 |
| **37** | 21205 | 21990 | - | 786 | Phage protein | 97 | 1E-176 | YP_002456002 |
| **38** | 22158 | 22580 | + | 423 | Phage protein | 99 | 5E-96 | YP_002456001 |
| **39** | 22567 | 23754 | + | 1188 | DNA helicase, phage-associated | 98 | 0 | YP_002456000 |
| **40** | 23915 | 24817 | + | 903 | Phage protein | 99 | 4E-68 | YP_007238220 |
| **41** | 24922 | 25923 | + | 1002 | Phage protein | 97 | 0 | YP_002455998 |
| **42** | 26012 | 26242 | + | 231 | Phage protein | 97 | 2E-35 | YP_001294481 |
| **43** | 26242 | 26460 | + | 219 | Phage protein | 99 | 3E-30 | YP_007002611 |
| **44** | 26444 | 26662 | + | 219 | Phage tail assembly | 99 | 1E-44 | YP_001294479 |
| **45** | 26662 | 26925 | + | 264 | Phage protein | 95 | 4E-52 | YP_001294478 |
| **46** | 26937 | 27143 | + | 207 | Phage protein | 96 | 9E-40 | YP_001294477 |
| **47** | 27143 | 28060 | + | 918 | Thymidylate synthase thyX | 97 | 0 | YP_002455991 |
| **48** | 28062 | 28253 | + | 192 | Phage protein | 98 | 2E-34 | YP_001294475 |
| **49** | 28256 | 29305 | + | 1050 | 3'-phosphatase, 5'-polynucleotide kinase, phage-associated | 89 | 0 | YP_002455989 |
| **50** | 29380 | 29934 | + | 555 | Phage protein | 100 | 4E-134 | YP_001294473 |
| **51** | 29934 | 33041 | + | 3108 | DNA polymerase III alpha subunit | 99 | 0 | YP_001294472 |
| **52** | 33034 | 33444 | + | 411 | Phage protein | 97 | 2E-78 | YP_002154288 |
| **53** | 33441 | 35000 | + | 1560 | DNA helicase, phage-associated | 99 | 0 | YP_001294470 |
| **54** | 35083 | 35715 | + | 633 | Phage protein | 100 | 5E-113 | YP_002455984 |
| **55** | 35804 | 36700 | + | 897 | Phage protein | 100 | 0E+00 | YP_002154194 |
| **56** | 36757 | 37362 | + | 606 | Phage protein | 100 | 2E-100 | YP_001294467 |
| **57** | 37359 | 37928 | + | 570 | Phage DNA-binding protein | 88 | 3E-114 | YP_002154192 |
| **58** | 37983 | 38894 | + | 912 | DNA ligase, phage-associated | 97 | 0 | YP_002455980 |
| **59** | 39174 | 39425 | + | 252 | Phage protein | 100 | 2E-39 | YP_002154190 |
| **60** | 39453 | 40115 | - | 663 | Lytic enzyme | 99 | 1E-159 | YP_009124368 |
| **61** | 40115 | 40543 | - | 429 | Phage protein | - | - | not available |
| **62** | 40546 | 43440 | - | 2895 | Phage tail fibers | - | - | not available |
| **63** | 43445 | 44959 | - | 1515 | Phage protein | - | - | not available |
| **64** | 44956 | 46209 | - | 1254 | Phage protein | - | - | not available |
| **65** | 46266 | 46697 | - | 432 | Phage baseplate | - | - | not available |
| **66** | 46677 | 46916 | + | 240 | hypothetical protein | - | - | not available |
| **67** | 46987 | 47520 | - | 534 | Phage protein | - | - | not available |
| **68** | 47520 | 48383 | - | 864 | Phage protein | - | - | not available |
| **69** | 48383 | 50965 | - | 2583 | Phage internal (core) protein | - | - | not available |
| **70** | 50969 | 51397 | - | 429 | Phage protein | - | - | not available |
| **71** | 51407 | 52000 | - | 594 | Phage tail fiber protein | - | - | not available |
| **72** | 52009 | 52413 | - | 405 | Phage protein | - | - | not available |
| **73** | 52548 | 53051 | - | 504 | Phage protein | - | - | not available |
| **74** | 53061 | 53489 | - | 429 | Phage protein | - | - | not available |
| **75** | 53491 | 53841 | - | 351 | Phage protein | - | - | not available |
| **76** | 53838 | 54161 | - | 324 | Phage protein | - | - | not available |
| **77** | 54161 | 54613 | - | 453 | Phage tail fiber protein | - | - | not available |
| **78** | 54671 | 56185 | - | 1515 | Phage protein | - | - | not available |
| **79** | 56780 | 57331 | - | 552 | Phage protein | - | - | not available |
| **80** | 57339 | 57737 | - | 399 | Phage protein | - | - | not available |
| **81** | 57734 | 58201 | - | 468 | Phage protein | 100 | 1E-76 | YP_001294442 |
| **82** | 58216 | 58653 | - | 438 | Phage protein | 100 | 4E-103 | YP_001294441 |
| **83** | 58755 | 59903 | - | 1149 | Phage capsid and scaffold | 99 | 0 | YP_001294440 |
| **84** | 59913 | 60548 | - | 636 | Phage protein | 99 | 6E-145 | YP_002455953 |
| **85** | 60552 | 61979 | - | 1428 | Phage protein | 97 | 0E+00 | YP_002455952 |
| **86** | 62492 | 62632 | - | 141 | Phage protein | 93 | 2E-19 | YP_002455951 |
| **87** | 62629 | 62835 | - | 207 | Phage protein | 97 | 1E-38 | YP_001294436 |
| **88** | 62855 | 63691 | - | 837 | Phage minor capsid protein | 98 | 0 | YP_001294435 |
| **89** | 63691 | 65988 | - | 2298 | Phage-related protein | 99 | 0 | YP_001294434 |

**Table S4:** General features of putative ORFs from phage DL62 with best matches in the NCBI database.

| **ORF** | **bp** | | **Strand** | **Gene Length** | **Representative similarity to proteins in database** | **% Identity** | **E-value** | **Accession no.** |
| --- | --- | --- | --- | --- | --- | --- | --- | --- |
|  | **Start** | **Stop** |  |  |  |  |  |  |
| **1** | 160 | 369 | - | 210 | hypothetical protein | 53 | 3 | XP_005307517 |
| **2** | 577 | 861 | + | 285 | hypothetical protein | - | - | not available |
| **3** | 1769 | 2053 | + | 285 | Phage protein | 100 | 0 | NP_877440 |
| **4** | 2053 | 2280 | + | 228 | Phage protein | 97 | 0 | NP_877441 |
| **5** | 2291 | 2830 | + | 540 | Phage protein | 100 | 0 | YP_001671945 |
| **6** | 2999 | 3118 | + | 120 | Phage protein | 97 | 0 | AIK67566 |
| **7** | 3197 | 3565 | + | 369 | Phage protein | 100 | 0 | YP_001671946 |
| **8** | 3552 | 3779 | + | 228 | Phage protein | 96 | 0 | YP_009125704 |
| **9** | 3958 | 4131 | + | 174 | Phage protein | 72 | 0 | YP_002117787 |
| **10** | 4131 | 4412 | + | 282 | Phage protein | 76 | 0 | YP_008431317 |
| **11** | 4412 | 4672 | + | 261 | Phage protein | 60 | 0 | YP_002727828 |
| **12** | 4674 | 4961 | + | 288 | Phage protein | 98 | 0 | YP_001671951 |
| **13** | 5040 | 5459 | + | 420 | Phage tail fibers | 98 | 0 | YP_001522798 |
| **14** | 5528 | 5887 | + | 360 | Phage protein | 100 | 0 | YP_001671953 |
| **15** | 5890 | 6615 | + | 726 | Phage DNA-binding protein | 60 | 0 | YP_001671954 |
| **16** | 6695 | 7114 | + | 420 | Phage protein | 96 | 0 | YP_656507 |
| **17** | 7080 | 7427 | + | 348 | Phage protein | 97 | 0 | YP_002117736 |
| **18** | 7417 | 7545 | + | 129 | Phage protein | 100 | 0 | YP_001522803 |
| **19** | 7613 | 8437 | + | 825 | DNA primase/helicase, phage-associated | 99 | 0 | YP_001671958 |
| **20** | 8406 | 9674 | + | 1269 | DNA helicase, phage-associated | 100 | 0 | YP_009125717 |
| **21** | 9664 | 10275 | + | 612 | Putative protein | - | - | not available |
| **22** | 10275 | 10772 | + | 498 | DNA ligase, phage-associated | - | - | not available |
| **23** | 10793 | 11221 | + | 429 | DNA ligase, phage-associated | - | - | not available |
| **24** | 11224 | 11553 | + | 330 | Phage protein | - | - | not available |
| **25** | 11550 | 13976 | + | 2427 | DNA polymerase I, phage-associated | - | - | not available |
| **26** | 14032 | 15081 | + | 1050 | Phage protein | - | - | not available |
| **27** | 15081 | 16022 | + | 942 | Phage protein | - | - | not available |
| **28** | 16012 | 16452 | + | 441 | Phage endonuclease | - | - | not available |
| **29** | 16449 | 17495 | + | 1047 | Phage exonuclease | - | - | not available |
| **30** | 17505 | 17876 | + | 372 | Phage protein | - | - | not available |
| **31** | 17869 | 18219 | + | 351 | Phage protein | - | - | not available |
| **32** | 18228 | 20675 | + | 2448 | Phage RNA polymerase | - | - | not available |
| **33** | 20849 | 21100 | + | 252 | Phage protein | - | - | not available |
| **34** | 21100 | 21573 | + | 474 | Putative protein | - | - | not available |
| **35** | 21539 | 21814 | + | 276 | Phage protein | - | - | not available |
| **36** | 21826 | 23358 | + | 1533 | Phage collar | - | - | not available |
| **37** | 23362 | 24330 | + | 969 | Phage capsid and scaffold | - | - | not available |
| **38** | 24383 | 25390 | + | 1008 | Phage capsid and scaffold | - | - | not available |
| **39** | 25487 | 26041 | + | 555 | Phage tail fibers | - | - | not available |
| **40** | 26044 | 28524 | + | 2481 | Phage tail fibers | - | - | not available |
| **41** | 28524 | 29069 | + | 546 | Phage internal (core) protein | 99 | 0 | AIK67602 |
| **42** | 29069 | 31765 | + | 2697 | Phage baseplate hub; Phage lysozyme | 100 | 0 | YP_008431349 |
| **43** | 31769 | 35782 | + | 4014 | Phage internal (core) protein | 99 | 0 | YP_009125740 |
| **44** | 35784 | 36539 | + | 756 | Phage tail fibers | 100 | 0 | YP_001671983 |
| **45** | 36539 | 36997 | + | 459 | Phage protein | 99 | 0 | YP_002117765 |
| **46** | 36990 | 37898 | + | 909 | Phage protein | 98 | 0 | YP_009125743 |
| **47** | 37902 | 38507 | + | 606 | Phage protein | 100 | 0 | YP_009125744 |
| **48** | 38507 | 38812 | + | 306 | Phage protein | 100 | 0 | YP_001522834 |
| **49** | 38822 | 40627 | + | 1806 | Phage terminase, large subunit (T7-like direct terminal repeats) | 100 | 0 | YP_009125746 |
| **50** | 40624 | 40824 | + | 201 | Phage protein | 98 | 0 | NP_877483 |
| **51** | 40821 | 41303 | + | 483 | Phage lysin | 99 | 0 | YP_008431358 |
| **52** | 41261 | 41590 | + | 330 | Phage protein | 98 | 0 | YP_008431359 |
| **53** | 41565 | 41678 | + | 114 | Phage protein | 100 | 0 | YP_009125750 |
| **54** | 41680 | 42093 | + | 414 | Phage protein | 98 | 0 | YP_009125751 |
| **55** | 42112 | 42357 | + | 246 | Phage protein | 96 | 0 | YP_001671994 |

**Table S5:** General features of putative ORFs from phage DL64 with best matches in the NCBI database.

| **ORF** | **bp** | | **Strand** | **Gene Length** | **Representative similarity to proteins in database** | **% Identity** | **E-value** | **Accession no.** |
| --- | --- | --- | --- | --- | --- | --- | --- | --- |
|  | **Start** | **Stop** |  |  |  |  |  |  |
| **1** | 44 | 772 | + | 729 | N4 gp69-like protein | - | - | not available |
| **2** | 769 | 2421 | + | 1653 | Phage protein | - | - | not available |
| **3** | 2418 | 3152 | + | 735 | N4 gp67-like protein | - | - | not available |
| **4** | 3184 | 3483 | - | 300 | hypothetical protein | - | - | not available |
| **5** | 3487 | 3873 | - | 387 | Deoxyuridine 5'-triphosphate nucleotidohydrolase | - | - | not available |
| **6** | 3941 | 6121 | + | 2181 | Phage portal protein | - | - | not available |
| **7** | 6191 | 6529 | + | 339 | hypothetical protein | - | - | not available |
| **8** | 6529 | 7731 | + | 1203 | MJ0042 family finger-like protein | - | - | not available |
| **9** | 7766 | 8965 | + | 1200 | Phage protein | - | - | not available |
| **10** | 9022 | 9687 | + | 666 | Phage protein | - | - | not available |
| **11** | 9880 | 10656 | + | 777 | N4 gp54-like protein | - | - | not available |
| **12** | 10713 | 12935 | + | 2223 | Phage protein | - | - | not available |
| **13** | 12916 | 13383 | + | 468 | N4 gp52-like protein | - | - | not available |
| **14** | 13383 | 14948 | + | 1566 | hypothetical protein | - | - | not available |
| **15** | 14949 | 25145 | + | 10197 | Phage protein | - | - | not available |
| **16** | 25161 | 25364 | - | 204 | hypothetical protein | - | - | not available |
| **17** | 25374 | 25691 | - | 318 | hypothetical protein | - | - | not available |
| **18** | 25688 | 25924 | - | 237 | hypothetical protein | - | - | not available |
| **19** | 26329 | 26733 | - | 405 | hypothetical protein | - | - | not available |
| **20** | 26791 | 27228 | - | 438 | hypothetical protein | - | - | not available |
| **21** | 27240 | 27800 | - | 561 | hypothetical protein | 100 | 9E-126 | YP_003358462 |
| **22** | 27788 | 28231 | - | 444 | Phage protein | 100 | 1E-87 | YP_003358461 |
| **23** | 28228 | 28455 | - | 228 | hypothetical protein | 97 | 1E-45 | YP_003358460 |
| **24** | 28597 | 29343 | - | 747 | Phage protein | 100 | 2E-155 | YP_003358459 |
| **25** | 29370 | 30104 | - | 735 | Phage protein | 100 | 0 | AFO70558 |
| **26** | 30153 | 32321 | - | 2169 | DNA primase, phage associated | 100 | 0 | AFO70557 |
| **27** | 32336 | 33346 | - | 1011 | Phage protein | 100 | 0 | YP_003358456 |
| **28** | 33348 | 33635 | - | 288 | hypothetical protein | 99 | 1E-61 | YP_003358455 |
| **29** | 33725 | 34153 | - | 429 | hypothetical protein | 100 | 1E-98 | YP_003358454 |
| **30** | 34522 | 35835 | + | 1314 | Phage tail fiber protein | 100 | 0 | AIZ94898 |
| **31** | 35832 | 36131 | + | 300 | hypothetical protein | 97 | 1E-60 | AFO70552 |
| **32** | 36128 | 36799 | + | 672 | hypothetical protein | 99 | 2E-160 | AIZ94804 |
| **33** | 36838 | 40059 | + | 3222 | Putative tail fiber protein | 98 | 0 | YP_003358449 |
| **34** | 40366 | 40608 | + | 243 | hypothetical protein | 100 | 3E-48 | YP_003358447 |
| **35** | 40605 | 41129 | + | 525 | hypothetical protein | 100 | 7E-124 | YP_003358446 |
| **36** | 41126 | 41638 | + | 513 | hypothetical protein | 100 | 4E-106 | YP_003358445 |
| **37** | 41701 | 42255 | - | 555 | hypothetical protein | 100 | 1E-132 | YP_003358444 |
| **38** | 42248 | 42439 | - | 192 | hypothetical protein | 100 | 6E-37 | YP_003358443 |
| **39** | 42489 | 42689 | - | 201 | hypothetical protein | 98 | 3E-39 | AFO70544 |
| **40** | 42890 | 43114 | - | 225 | hypothetical protein | 99 | 3E-24 | AFO70543 |
| **41** | 43162 | 44901 | - | 1740 | Phage lysis inhibitor # T4-like rIIA-rIIB membrane associated #T4 GC1698 | - | - | not available |
| **42** | 44913 | 47429 | - | 2517 | Phage rIIA lysis inhibitor | - | - | not available |
| **43** | 47433 | 47624 | - | 192 | Phage protein | - | - | not available |
| **44** | 47621 | 48019 | - | 399 | hypothetical protein | - | - | not available |
| **45** | 48022 | 48162 | - | 141 | hypothetical protein | - | - | not available |
| **46** | 48194 | 48664 | - | 471 | dCMP deaminase, Late competence protein ComEB | - | - | not available |
| **47** | 48624 | 48848 | - | 225 | hypothetical protein | - | - | not available |
| **48** | 48845 | 51460 | - | 2616 | DNA polymerase I | - | - | not available |
| **49** | 51460 | 51987 | - | 528 | hypothetical protein | - | - | not available |
| **50** | 51987 | 53153 | - | 1167 | RecD-like DNA helicase YrrC | - | - | not available |
| **51** | 53243 | 54472 | - | 1230 | hypothetical protein | - | - | not available |
| **52** | 55001 | 56071 | - | 1071 | Phage protein | - | - | not available |
| **53** | 56103 | 56345 | - | 243 | hypothetical protein | - | - | not available |
| **54** | 56342 | 57175 | - | 834 | ATP-dependent Clp protease ATP-binding subunit ClpX | - | - | not available |
| **55** | 57203 | 57388 | - | 186 | hypothetical protein | - | - | not available |
| **56** | 57632 | 57856 | - | 225 | hypothetical protein | - | - | not available |
| **57** | 57853 | 58368 | - | 516 | hypothetical protein | - | - | not available |
| **58** | 58368 | 58949 | - | 582 | hypothetical protein | - | - | not available |
| **59** | 58949 | 59503 | - | 555 | hypothetical protein | - | - | not available |
| **60** | 59500 | 60126 | - | 627 | hypothetical protein | - | - | not available |
| **61** | 60130 | 60354 | - | 225 | hypothetical protein | 97 | 1E-16 | YP_003358421 |
| **62** | 60427 | 60579 | - | 153 | hypothetical protein | 100 | 1E-27 | AIZ94866 |
| **63** | 60677 | 61918 | - | 1242 | Phage protein | 100 | 0 | AIX13178 |
| **64** | 61953 | 62225 | - | 273 | hypothetical protein | 97 | 6E-43 | AFO70520 |
| **65** | 62258 | 62572 | - | 315 | hypothetical protein | 88 | 8E-61 | AIZ94863 |
| **66** | 62864 | 63124 | - | 261 | hypothetical protein | 95 | 2E-52 | AIZ94862 |
| **67** | 63137 | 64069 | - | 933 | RNA polymerase, phage-associated | 98 | 0 | AFO70518 |
| **68** | 64081 | 64428 | - | 348 | hypothetical protein | 100 | 5E-80 | AIZ94951 |
| **69** | 64468 | 64908 | - | 441 | hypothetical protein | 99 | 1E-103 | AIZ94950 |
| **70** | 64908 | 65159 | - | 252 | hypothetical protein | 98 | 8E-52 | AIZ94766 |
| **71** | 65159 | 65560 | - | 402 | Phage protein | 99 | 4E-94 | AIZ94765 |
| **72** | 65560 | 65895 | - | 336 | hypothetical protein | 85 | 9E-58 | AIZ94764 |
| **73** | 66080 | 66370 | - | 291 | hypothetical protein | 100 | 1E-60 | YP_009031789 |
| **74** | 66462 | 66647 | - | 186 | hypothetical protein | 98 | 8E-35 | YP_003358409 |
| **75** | 66719 | 66940 | - | 222 | hypothetical protein | 100 | 7E-43 | YP_003358408 |
| **76** | 66937 | 67170 | - | 234 | hypothetical protein | 100 | 2E-44 | AIZ94852 |
| **77** | 67167 | 67385 | - | 219 | hypothetical protein | 97 | 8E-30 | AFO70508 |
| **78** | 67415 | 67618 | - | 204 | hypothetical protein | 93 | 2E-33 | YP_003358405 |
| **79** | 67622 | 67924 | - | 303 | hypothetical protein | 88 | 5E-58 | YP_003358404 |
| **80** | 67908 | 68204 | - | 297 | hypothetical protein | 93 | 2E-62 | YP_003358403 |
| **81** | 68208 | 68459 | - | 252 | hypothetical protein | 96 | 3E-52 | AIZ94847 |
| **82** | 68459 | 68689 | - | 231 | hypothetical protein | 99 | 1E-46 | YP_003358401 |
| **83** | 68686 | 68928 | - | 243 | hypothetical protein | 90 | 2E-44 | YP_003358400 |
| **84** | 68943 | 69134 | - | 192 | hypothetical protein | 100 | 4E-39 | AIZ94844 |
| **85** | 69542 | 69796 | - | 255 | hypothetical protein | 99 | 4E-49 | YP_003358398 |
| **86** | 70216 | 70878 | - | 663 | hypothetical protein | 91 | 5E-132 | AFO70587 |
| **87** | 71081 | 71347 | - | 267 | hypothetical protein | 66 | 6E-19 | AFO70586 |
| **88** | 71455 | 71715 | - | 261 | hypothetical protein | 67 | 4E-06 | AIZ94839 |
| **89** | 71712 | 71924 | - | 213 | hypothetical protein | 99 | 1E-42 | YP_009031858 |
| **90** | 71924 | 72355 | - | 432 | hypothetical protein | 98 | 2E-97 | YP_003358483 |

**Table S6:** General features of putative ORFs from phage DL68 with best matches in the NCBI database.

| **ORF** | **bp** | | **Strand** | **Gene Length** | **Representative similarity to proteins in database** | **% Identity** | **E-value** | **Accession no.** |
| --- | --- | --- | --- | --- | --- | --- | --- | --- |
|  | **Start** | **Stop** |  |  |  |  |  |  |
| **1** | 66 | 467 | + | 402 | Phage protein | 100 | 0 | YP_002364325 |
| **2** | 636 | 821 | + | 186 | Phage protein | 98 | 0 | YP_002364324 |
| **3** | 818 | 1021 | + | 204 | Phage protein | 97 | 0 | YP_007002562 |
| **4** | 1027 | 1338 | + | 312 | Phage protein | 100 | 0 | YP_006200780 |
| **5** | 1587 | 1934 | + | 348 | Phage protein | 99 | 0 | YP_002364322 |
| **6** | 1954 | 2454 | + | 501 | Phage protein | 100 | 0 | YP_002364321 |
| **7** | 2557 | 3489 | + | 933 | Phage protein | 100 | 0 | YP_002364320 |
| **8** | 3591 | 4178 | + | 588 | Phage protein | 82 | 0 | YP_009009031 |
| **9** | 4195 | 4632 | + | 438 | Phage protein | 100 | 0 | YP_006200775 |
| **10** | 4719 | 5498 | + | 780 | Phage protein | 100 | 0 | YP_002364316 |
| **11** | 5501 | 5899 | + | 399 | Phage protein | 97 | 0 | YP_002154151 |
| **12** | 5943 | 6293 | + | 351 | Phage protein | 99 | 0 | YP_009124329 |
| **13** | 6293 | 6508 | + | 216 | Phage protein | 100 | 0 | YP_002418812 |
| **14** | 6508 | 6891 | + | 384 | Phage protein | 99 | 0 | YP_007002551 |
| **15** | 6928 | 8310 | - | 1383 | Phage terminase, large subunit | 100 | 0 | YP_002418810 |
| **16** | 8315 | 8437 | - | 123 | Phage protein | 78 | 0 | YP_002154146 |
| **17** | 8480 | 8770 | + | 291 | hypothetical protein | - | - | not available |
| **18** | 8800 | 8955 | + | 156 | hypothetical protein | 57 | 0 | ERY56378 |
| **19** | 9011 | 9313 | + | 303 | Phage protein | 98 | 0 | YP_002364309 |
| **20** | 9368 | 10054 | + | 687 | Phage capsid and scaffold | 99 | 0 | YP_006200858 |
| **21** | 10057 | 10245 | + | 189 | Phage protein | 88 | 0 | YP_006200857 |
| **22** | 10332 | 10583 | + | 252 | Phage protein | 100 | 0 | YP_006200856 |
| **23** | 10580 | 10780 | + | 201 | Phage protein | 100 | 0 | YP_002418895 |
| **24** | 10777 | 10992 | + | 216 | Phage protein | 99 | 0 | YP_006200854 |
| **25** | 10989 | 11198 | + | 210 | Phage protein | 100 | 0 | YP_002364393 |
| **26** | 11230 | 11874 | + | 645 | Phage protein | 100 | 0 | YP_006200852 |
| **27** | 11875 | 12204 | + | 330 | Phage protein | 97 | 0 | YP_002154316 |
| **28** | 12266 | 12490 | + | 225 | Phage protein | 97 | 0 | YP_009124310 |
| **29** | 12544 | 12765 | + | 222 | Phage protein | 100 | 0 | YP_002364388 |
| **30** | 12818 | 12946 | + | 129 | Phage protein | 98 | 0 | YP_002364387 |
| **31** | 12980 | 13129 | + | 150 | Phage protein | 98 | 0 | YP_002364387 |
| **32** | 13140 | 13835 | + | 696 | Phage protein | 100 | 0 | YP_002364386 |
| **33** | 14025 | 14636 | + | 612 | Phage protein | 100 | 0 | YP_002364385 |
| **34** | 14804 | 15373 | - | 570 | Phage protein | 100 | 0 | YP_002364384 |
| **35** | 16337 | 18076 | - | 1740 | DNA primase, phage associated | 99 | 0 | YP_009124396 |
| **36** | 18224 | 18409 | - | 186 | Phage protein | 100 | 0 | YP_001294490 |
| **37** | 18415 | 19491 | - | 1077 | Phage protein | 100 | 0 | YP_002364381 |
| **38** | 19488 | 19940 | - | 453 | Phage protein | 99 | 0 | YP_002364380 |
| **39** | 19964 | 21268 | - | 1305 | hypothetical protein | 100 | 0 | WP_023099019 |
| **40** | 21279 | 22064 | - | 786 | Phage protein | 100 | 0 | YP_002364378 |
| **41** | 22233 | 22655 | + | 423 | Phage protein | 100 | 0 | YP_001294485 |
| **42** | 22642 | 23829 | + | 1188 | DNA helicase, phage-associated | 100 | 0 | YP_002418875 |
| **43** | 23991 | 24875 | + | 885 | Phage protein | 99 | 0 | YP_002364375 |
| **44** | 24980 | 25981 | + | 1002 | Phage protein | 98 | 0 | YP_002418873 |
| **45** | 26069 | 26299 | + | 231 | Phage protein | 100 | 0 | YP_002364373 |
| **46** | 26299 | 26517 | + | 219 | Phage protein | 100 | 0 | YP_002154206 |
| **47** | 26501 | 26719 | + | 219 | Phage tail assembly protein | 100 | 0 | YP_002418870 |
| **48** | 26752 | 26982 | + | 231 | Phage protein | 100 | 0 | YP_002418869 |
| **49** | 26990 | 27196 | + | 207 | Phage protein | 100 | 0 | YP_002364369 |
| **50** | 27196 | 28113 | + | 918 | Thymidylate synthase thyX | 100 | 0 | YP_006200825 |
| **51** | 28115 | 28306 | + | 192 | Phage protein | 100 | 0 | YP_006200824 |
| **52** | 28309 | 29328 | + | 1020 | 3'-phosphatase, 5'-polynucleotide kinase, phage-associated | 96 | 0 | YP_007238211 |
| **53** | 29405 | 29959 | + | 555 | Phage protein | 100 | 0 | YP_007002604 |
| **54** | 29959 | 33066 | + | 3108 | DNA polymerase III alpha subunit | 99 | 0 | YP_006200821 |
| **55** | 33059 | 33469 | + | 411 | Phage protein | 100 | 0 | YP_007002602 |
| **56** | 33466 | 35025 | + | 1560 | DNA helicase, phage-associated | 100 | 0 | YP_002364362 |
| **57** | 35120 | 35740 | + | 621 | Phage protein | 100 | 0 | YP_002154286 |
| **58** | 35829 | 36728 | + | 900 | Phage protein | 100 | 0 | YP_006200817 |
| **59** | 36782 | 37387 | + | 606 | Phage protein | 100 | 0 | YP_001294467 |
| **60** | 37384 | 37938 | + | 555 | Phage DNA binding protein | 99 | 0 | YP_006200815 |
| **61** | 37993 | 38904 | + | 912 | DNA ligase, phage-associated | 100 | 0 | YP_002154282 |
| **62** | 39184 | 39435 | + | 252 | Phage protein | 100 | 0 | YP_002364356 |
| **63** | 39460 | 40122 | - | 663 | Lytic enzyme | 100 | 0 | YP_006200812 |
| **64** | 40122 | 40550 | - | 429 | Phage protein | 99 | 0 | YP_002154188 |
| **65** | 40552 | 43446 | - | 2895 | Phage tail fibers | 98 | 0 | YP_009124366 |
| **66** | 43451 | 44965 | - | 1515 | Phage protein | 97 | 0 | YP_006200809 |
| **67** | 44962 | 46215 | - | 1254 | Phage protein | 100 | 0 | YP_009008971 |
| **68** | 46272 | 46835 | - | 564 | Phage baseplate | 99 | 0 | YP_001294458 |
| **69** | 46993 | 47526 | - | 534 | Phage protein | 99 | 0 | YP_002364349 |
| **70** | 47526 | 48389 | - | 864 | Phage protein | 100 | 0 | YP_002154273 |
| **71** | 48389 | 50965 | - | 2577 | Phage internal (core) protein | 100 | 0 | YP_002154272 |
| **72** | 50969 | 51397 | - | 429 | Phage protein | 100 | 0 | YP_002154180 |
| **73** | 51407 | 52000 | - | 594 | Phage tail fiber protein | 100 | 0 | YP_002154179 |
| **74** | 52009 | 52413 | - | 405 | Phage protein | 100 | 0 | YP_002154269 |
| **75** | 52548 | 53051 | - | 504 | Phage protein | 100 | 0 | YP_002418842 |
| **76** | 53061 | 53492 | - | 432 | Phage protein | 98 | 0 | YP_002418841 |
| **77** | 53494 | 53844 | - | 351 | Phage protein | 100 | 0 | YP_002154266 |
| **78** | 53841 | 54164 | - | 324 | Phage protein | 100 | 0 | YP_002154265 |
| **79** | 54164 | 54616 | - | 453 | Phage tail fiber protein | 100 | 0 | YP_002154264 |
| **80** | 54675 | 56189 | - | 1515 | Phage protein | 100 | 0 | YP_007002577 |
| **81** | 56205 | 56729 | - | 525 | Phage protein | 100 | 0 | YP_007238183 |
| **82** | 56783 | 57334 | - | 552 | Phage protein | 100 | 0 | YP_002154170 |
| **83** | 57342 | 57530 | - | 189 | Phage protein | 100 | 0 | YP_002364335 |
| **84** | 57737 | 58204 | - | 468 | Phage protein | 99 | 0 | YP_002154168 |
| **85** | 58219 | 58656 | - | 438 | Phage protein | 100 | 0 | YP_002154167 |
| **86** | 58758 | 59906 | - | 1149 | Phage capsid and scaffold | 100 | 0 | YP_002364332 |
| **87** | 59916 | 60551 | - | 636 | Phage protein | 100 | 0 | YP_002364331 |
| **88** | 60555 | 61988 | - | 1434 | Phage protein | 100 | 0 | YP_002364330 |
| **89** | 62502 | 62642 | - | 141 | Phage protein | 100 | 0 | YP_002418828 |
| **90** | 62639 | 62845 | - | 207 | Phage protein | 100 | 0 | YP_002418827 |
| **91** | 62864 | 63700 | - | 837 | Phage minor capsid protein | 100 | 0 | YP_002364327 |
| **92** | 63700 | 65997 | - | 2298 | Phage-related protein | 99 | 0 | YP_002364326 |
